# Codon usage patterns in Nematoda: analysis based on over 25 million codons in thirty-two species

**DOI:** 10.1186/gb-2006-7-8-r75

**Published:** 2006-08-14

**Authors:** Makedonka Mitreva, Michael C Wendl, John Martin, Todd Wylie, Yong Yin, Allan Larson, John Parkinson, Robert H Waterston, James P McCarter

**Affiliations:** 1 Genome Sequencing Center, Washington University School of Medicine, St Louis, Missouri 63108, USA; 2 Department of Biology, Washington University, St. Louis, Missouri 63130, USA; 3 Hospital for Sick Children, Toronto, and Departments of Biochemistry/Medical Genetics and Microbiology, University of Toronto, M5G 1X8, Canada; 4 Department of Genome Sciences, University of Washington, Seattle, Washington 98195, USA; 5 Divergence Inc., St Louis, Missouri 63141, USA

## Abstract

**Background:**

Codon usage has direct utility in molecular characterization of species and is also a marker for molecular evolution. To understand codon usage within the diverse phylum Nematoda, we analyzed a total of 265,494 expressed sequence tags (ESTs) from 30 nematode species. The full genomes of *Caenorhabditis elegans *and *C. briggsae *were also examined. A total of 25,871,325 codons were analyzed and a comprehensive codon usage table for all species was generated. This is the first codon usage table available for 24 of these organisms.

**Results:**

Codon usage similarity in Nematoda usually persists over the breadth of a genus but then rapidly diminishes even within each clade. *Globodera*, *Meloidogyne*, *Pristionchus*, and *Strongyloides *have the most highly derived patterns of codon usage. The major factor affecting differences in codon usage between species is the coding sequence GC content, which varies in nematodes from 32% to 51%. Coding GC content (measured as GC3) also explains much of the observed variation in the effective number of codons (R = 0.70), which is a measure of codon bias, and it even accounts for differences in amino acid frequency. Codon usage is also affected by neighboring nucleotides (N1 context). Coding GC content correlates strongly with estimated noncoding genomic GC content (R = 0.92). On examining abundant clusters in five species, candidate optimal codons were identified that may be preferred in highly expressed transcripts.

**Conclusion:**

Evolutionary models indicate that total genomic GC content, probably the product of directional mutation pressure, drives codon usage rather than the converse, a conclusion that is supported by examination of nematode genomes.

## Abstract 2

A codon usage table for 32 nematode species is presented and suggests that total genomic GC content drives codon usage.

## Background

Utilization of the degenerate triplet code for amino acid (AA) translation is neither uniform nor random. In particular, there are distinct patterns among different species and genes. Such patterns can readily be characterized by codon usage, namely the observed percentage occurrence with which each codon is used to encode a given AA. This measure has direct utility in molecular characterization of a species in that it enables efficient degenerate and nondegenerate primer design for cross-species gene cloning, open reading frame determination, and optimal protein expression [1]. Such tools are particularly important with respect to species for which limited molecular information exists. Codon usage also serves as an indicator of molecular evolution [2]. Codon usage bias, namely the degree to which usage departs from uniform use of all available codons for an AA, can be influenced by a number of evolutionary processes. The guanine and cytosine (GC) versus adenine and thymine (AT) composition of the species' genome, probably the product of directional mutation pressure [3,4], is a key driver of both codon usage and AA composition [5,6]. Other factors that influence codon usage may include the relative abundance of isoaccepting tRNAs [7-9], especially for highly expressed mRNAs that require translational efficiency [10,11], presence of mRNA secondary structure [12,13], and facilitation of correct co-translational protein folding [14]. Codon usage appears not to be optimized to minimize the impact of errors in translation and replication [15].

Nematodes are a highly abundant and diverse group of organisms that exploit niches from free-living microbivory to plant and animal parasitism. Molecular phylogenies divide nematodes into five major named and numbered clades within which parasitism has arisen multiple times [16]: Dorylaimia (clade I), Enoplia (clade II), Spirurina (clade III), Tylenchina (clade IV), and Rhabditina (clade V). Following the sequencing of the complete genome of the model nematode *Caenorhabditis elegans *[17], we have begun to catalog the molecular diversity of nematode genomes through the generation of over 250,000 expressed sequence tags (ESTs) from more than 30 nematode species (including 28 parasites) in four clades. Gene expression analyses for several medically and economically important parasites such as filarial, hookworm, and root knot nematode species have been completed [18-23] (for reviews [24,25]). Moreover, we recently conducted a meta-analysis of partial genomes across the whole phylum with a focus on the conservation and diversification of encoded protein families [26]. Project information is maintained on several online resources [27-30].

Now, in the most extensive such study yet performed for any phylum, we extend the above analyses with a comprehensive survey of observed codon usage and bias based on nearly 26 million codons in 32 species of the Nematoda. Because of its completed genome, *C. elegans *has been the primary species utilized in nematode codon usage studies [31-34]. Our findings provide more complete information for *Caenorhabditis *based on all 41,782 currently predicted proteins in *C. elegans *and *C. briggsae *[35]. Studies for other nematode species have been more limited. Codon usage has been tabulated for a number of parasitic nematodes including filarial species *Brugia malayi*, *Onchocerca volvulus*, *Wucheria bancrofti*, *Acanthocheilonema viteae*, *Dirofilaria immitis *[36-39], *Strongyloides stercoralis *[40], *Ascaris suum *[41], *Ancylostoma caninum*, and *Necator americanus *[42]. Although Fadiel and coworkers [39] used up to 60 genes per species, sample sizes in the other studies were quite small, typically fewer than 10 representative genes and 5,000 codons per species. In the present study we used an average of 2,350 genes and 270,000 codons per species for the 30 non-*Caenorhabditis *species. Our results provide the first codon usage tables for 24 of these organisms. Web available automated codon usage databases compiled from GenBank [43] lack almost all of this information because they rely only on full-length protein coding gene sequence submissions rather than the EST data used here.

In analyzing codon distribution in Nematoda, we describe how average usage varies between species and across the phylum. For instance, it has been shown that there is a level of conservation in codon distribution between 'closely' related nematodes such as *Brugia malayi *and *B. pahangi *[37] and *Brugia *and *Onchocerca *[38]. These relationships do not appear to extend over greater evolutionary distances, for instance between *Onchocerca *and *Caenorhabditis *[36]. The evolutionary distance at which conservation of codon usage diminishes has not previously been established [32]. Here we show that codon usage similarity in Nematoda is a relatively short-range phenomenon, generally persisting over the breadth of a genus but then rapidly diminishing within each clade. We also show that the major factor affecting differences in mean codon usage between distantly related species is the coding sequence GC as compared with AT content. GC content also explains much of the observed variation in the effective number of codons, a measure of codon bias, and even differences in AA frequency.

## Results

### Determination of codon usage patterns and amino acid composition

Extensive nucleotide sequence data are now available for many nematode species, largely because of recent progress using genomic approaches [25,44]. To obtain a better understanding of codon usage and AA composition within the phylum Nematoda, we analyzed a total of 265,494 EST sequences originating from 30 nematode species. The ESTs define 93,645 clusters or putative genes, with 208-9,511 clusters per species (Table 1) [26]. Table 1 also provides two letter codes for the nematode species used throughout the remainder of the report. We used prot4EST, a translation prediction pipeline optimized for EST datasets [45], to generate protein predictions. To reduce noise derived from poor translations, our analysis considered only the longest open reading frame (ORF) translations with strong supporting evidence in the form of similarity to known or predicted proteins (BLASTX cutoff 1 × e^-8^) and retained only the polypeptide aligned portion of the nucleotide sequence. About 75% of the clusters met these criteria, yielding 8,080,057 codons originating from species other than *Caenorhabditis*, and 25,871,325 total codons from all 32 species including available predictions from *C. elegans *and *C. briggsae*. The 18 AA residues with redundant codons gave a total of (18) × C_32,2 _= 496 comparisons of codon usage between species. Comprehensive tables of AA composition (Tables 2 and 3) and codon usage (Table 4) for all 32 Nematoda species studied are provided. Below we use these tables to examine, first, variation in AA composition and its relationship to GC content and, second, codon usage and its relationship to GC content.

**Table 1:** Summary of sequences used by nematode species

Clade	Code	Species	ESTs	Total number of clusters	Clusters or genes used		Codons	GC content (%)
							
					*n*	%		
V	*NA*	*Necator americanus*^a^	4,766	2,294	1,784	78	192,756	46
	*AC*	*Ancylostoma caninum*^b^	9,079	4,203	3,207	76	305,036	48
	*AY*	*Ancylostoma ceylanicum*^b^	10,544	3,485	2,814	81	387,372	49
	*NB*	*Nippostrongylus brasiliensis*^b^	1,234	742	630	85	75,934	50
	*HC*	*Haemonchus contortus*^b^	17,268	4,146	4,102	99	584,513	47
	*OO*	*Ostertagia ostertagi*^b^	6,670	2,355	1,961	83	222,616	48
	*TD*	*Teladorsagia circumcincta*^b^	4,313	1,655	1,616	98	194,351	48
	*CE*	*Caenorhabditis elegans*^c^	-	-	22,254	100	9,784,215	43
	*CB*	*Caenorhabditis briggsae*^c^	-	-	19,528	100	8,007,053	44
	*PP*	*Pristionchus pacificus*^c^	8,672	3,690	2,597	70	297,605	51
IVa	SS	*Strongyloides stercoralis*^a^	11,236	3,635	2,803	77	367,308	33
	*SR*	*Strongyloides ratti*^b^	9,932	3,264	2,682	82	320,874	32
	*PT*	*Parastrongyloides trichosuri*^b^	7,712	3,086	2,457	80	284,785	40
IVb	PE	*Pratylenchus penetrans*^d^	1,908	408	338	83	45,802	46
	*GP*	*Globodera pallida*^d^	1,317	977	479	49	65,699	51
	*GR*	*Globodera rostochiensis*^d^	5,905	2,851	2,192	77	290,614	51
	*HG*	*Heterodera glycines*^d^	18,524	7,198	5,564	77	742,990	50
	*MI*	*Meloidogyne incognita*^d^	12,394	4,408	3,214	73	366,435	37
	*MJ*	*Meloidogyne javanica*^d^	5,282	2,609	2,086	80	203,135	36
	*MA*	*Meloidogyne arenaria*^d^	3,251	1,892	1,483	78	176,816	36
	*MH*	*Meloidogyne hapla*^d^	13,462	4,479	3,507	78	407,985	36
	*MC*	*Meloidogyne chitwoodi*^d^	7,036	2,409	1,906	79	205,612	35
	*ZP*	*Zeldia punctata*^c^	388	208	102	49	16,723	43
III	AS	*Ascaris suum*^b^	38,944	8,482	5,830	69	646,740	46
	*AL*	*Ascaris lumbricoides*^a^	1,822	853	508	60	42,919	47
	*TC*	*Toxocara canis*^b^	4,206	1,447	866	60	103,065	48
	*BM*	*Brugia malayi*^a^	25,067	9,511	6,483	68	561,296	39
	*DI*	*Dirofiliaria immitis*^b^	3,585	1,747	1,380	79	126,880	38
	*OV*	*Onchocerca volvulus*^a^	14,922	5,097	2,914	57	299,336	40
I	TS	*Trichinella spiralis*^a^	10,384	3,680	2,693	73	290,794	41
	*TM*	*Trichuris muris*^b^	2,713	1,577	1,179	75	147,995	49
	*TV*	*Trichuris vulpis*^b^	2,958	1,257	1,000	80	106,071	48

^a^Human parasite, ^b^animal parasite, ^c^free-living, and ^d^plant parasite. EST, expressed sequence tag.

**Table 2:** Amino acid composition (%) of translations by nematode species

Clade	Species	Amino acid																			
		
		A	C	D	E	F	G	H	I	K	L	M	N	P	Q	R	S	T	V	W	Y
		Ala	Cys	Asp	Glu	Phe	Gly	His	Ile	Lys	Leu	Met	Asn	Pro	Gln	Arg	Ser	Thr	Val	Typ	Tyr
V	NA	6.9	2.4	5.2	6.2	4.7	6.3	2.5	5.5	6.5	8.6	2.6	4.2	5.0	3.7	6.2	7.2	5.3	6.5	1.2	3.3
	AC	7.0	2.4	5.0	6.0	4.7	5.9	2.7	5.6	6.3	8.9	2.8	4.2	4.6	3.7	6.2	7.4	5.4	6.6	1.3	3.3
	AY	7.6	2.2	5.5	6.6	4.2	6.5	2.5	5.2	6.4	8.5	2.5	4.0	5.1	3.8	6.1	7.1	5.3	6.7	1.2	3.0
	NB	7.8	2.2	5.4	6.3	4.0	6.9	2.4	5.0	7.2	8.1	2.6	4.0	4.9	3.5	6.4	7.0	5.2	6.9	1.1	3.1
	HC	7.4	2.3	5.5	6.5	4.3	6.6	2.5	5.4	7.0	8.4	2.5	4.1	4.9	3.7	6.0	6.4	5.2	6.8	1.2	3.4
	OO	7.2	2.3	5.3	6.3	4.4	6.7	2.6	5.3	6.7	8.4	2.6	4.0	5.2	3.8	6.1	6.8	5.3	6.7	1.1	3.1
	TD	7.5	2.7	5.2	6.1	4.3	6.7	2.6	5.0	6.6	8.4	2.7	4.2	5.1	3.8	5.8	7.1	5.3	6.5	1.2	3.3
	CE	6.3	2.0	5.3	6.5	4.8	5.4	2.3	6.1	6.4	8.6	2.6	4.9	4.9	4.2	5.2	8.1	5.9	6.2	1.1	3.1
	CB	6.3	2.0	5.3	6.8	4.7	5.4	2.3	6.0	6.4	8.5	2.6	4.8	5.0	4.2	5.4	8.0	5.8	6.1	1.1	3.1
	PP	7.4	1.9	5.4	6.9	4.0	6.6	2.5	5.3	6.6	8.4	2.6	3.9	5.1	3.4	6.4	7.6	5.4	6.4	1.2	3.0
IVa	SS	5.5	1.9	5.7	7.0	4.3	6.0	2.1	7.1	8.0	8.3	2.3	6.0	4.5	3.7	4.6	7.2	5.5	5.8	1.0	3.5
	SR	5.4	2.0	5.4	6.5	4.7	5.9	2.1	7.4	8.1	8.6	2.4	6.2	4.3	3.6	4.3	7.2	5.4	5.8	1.0	3.8
	PT	6.3	2.0	5.3	6.3	4.6	6.3	2.4	6.6	8.1	8.3	2.4	5.3	4.3	3.5	4.9	6.9	5.5	6.1	1.0	3.7
IVb	PE	6.9	2.0	5.3	6.7	4.3	7.0	2.4	5.8	7.6	8.4	2.6	4.5	4.5	4.5	6.2	6.4	4.9	5.9	1.2	2.9
	GP	6.8	2.4	4.5	5.4	5.6	7.2	2.4	4.9	7.1	9.1	2.3	3.9	5.8	3.8	6.7	7.1	4.8	6.0	1.2	2.9
	GR	7.4	2.1	4.9	6.0	4.8	6.5	2.6	5.2	5.9	9.3	2.5	4.3	5.0	4.4	6.5	7.2	5.2	6.3	1.2	2.7
	HG	7.3	2.2	4.9	6.2	5.1	6.3	2.6	5.2	6.0	9.2	2.4	4.5	5.0	4.5	6.3	7.3	5.1	6.2	1.2	2.5
	MI	5.7	2.0	4.8	6.7	5.1	5.6	2.2	6.5	7.3	9.4	2.3	5.6	4.5	4.6	5.3	7.5	5.2	5.4	1.1	3.0
	MJ	5.5	2.1	4.8	6.6	5.5	5.4	2.2	6.9	7.8	9.6	2.4	5.6	4.2	4.2	5.4	7.0	5.1	5.4	1.1	3.3
	MA	5.7	2.0	5.0	6.9	5.3	5.7	2.2	6.9	7.3	9.7	2.3	5.5	4.1	4.3	5.2	7.0	5.0	5.6	1.1	3.2
	MH	5.6	2.0	4.9	6.8	5.3	5.6	2.2	7.0	7.3	9.5	2.3	5.8	4.3	4.3	5.2	7.3	4.9	5.4	1.2	3.2
	MC	5.3	2.3	4.8	6.4	5.5	5.5	2.3	7.4	7.4	9.7	2.3	6.0	4.0	4.3	4.9	7.4	5.0	5.2	1.1	3.3
	ZP	7.6	1.5	5.2	6.2	4.2	7.1	2.5	6.1	7.9	8.4	1.8	4.7	4.6	3.8	6.0	5.4	5.5	6.6	1.1	3.7
III	AS	7.0	2.5	4.9	6.1	4.6	5.8	2.5	6.0	6.3	8.7	2.6	4.5	4.8	3.6	6.3	7.5	5.3	6.6	1.2	3.3
	AL	7.3	2.6	4.6	5.8	4.9	6.1	2.5	6.0	6.4	8.3	2.5	4.3	5.3	3.5	6.2	7.4	5.2	6.5	1.2	3.4
	TC	7.3	2.8	5.0	6.0	4.2	6.8	2.6	5.3	7.3	8.0	2.4	4.1	5.4	3.5	6.2	6.7	5.6	6.4	1.2	3.2
	BM	5.6	2.5	4.6	5.6	5.4	5.0	2.6	7.1	6.8	9.6	2.8	5.0	4.1	3.8	5.6	7.8	5.3	5.9	1.1	3.6
	DI	5.6	2.4	4.9	6.0	5.2	4.7	2.7	7.5	7.0	9.5	2.7	5.2	3.9	3.8	5.9	7.5	5.1	5.8	1.1	3.8
	OV	6.0	2.2	5.0	6.1	4.9	5.6	2.5	6.9	7.1	8.9	2.7	4.9	4.5	3.9	5.9	7.3	5.2	5.7	1.2	3.5
I	TS	6.2	2.6	5.0	6.2	5.1	5.1	2.5	6.1	6.6	9.5	2.6	4.9	4.1	3.9	5.6	7.6	5.1	6.5	1.2	3.4
	TM	7.1	3.0	5.0	6.0	4.5	6.5	2.5	5.0	6.3	8.9	2.6	4.0	5.0	3.8	6.2	7.4	5.2	6.7	1.2	3.2
	TV	7.0	3.0	4.9	6.1	4.5	5.8	2.5	5.1	6.4	9.0	2.6	4.2	4.8	3.8	6.1	7.5	5.5	6.8	1.3	3.2

Definitions of species two letter codes are provided in Table 1.

**Table 3:** Amino acid composition (%) of translations from Nematoda and four reference species

Amino acid		Nematode		HS	DM	SC	EC
						
		Mean	SD				
A	Ala	6.6	0.8	7.1	7.5	5.6	9.2
C	Cys	2.3	0.3	2.3	1.9	1.3	1.1
D	Asp	5.1	0.3	4.8	5.2	5.8	5.2
E	Glu	6.3	0.4	6.9	6.4	6.5	5.7
F	Phe	4.7	0.5	3.8	3.5	4.4	3.8
G	Gly	6.1	0.7	6.6	6.3	5.1	7.3
H	His	2.4	0.2	2.6	2.7	2.2	2.2
I	Ile	6.0	0.8	4.4	4.9	6.5	6.0
K	Lys	6.9	0.6	5.6	5.6	7.3	4.8
L	Leu	8.8	0.5	10.0	9.0	9.5	10.1
M	Met	2.5	0.2	2.2	2.4	2.1	2.6
N	Asn	4.7	0.7	3.6	4.7	6.1	4.3
P	Pro	4.7	0.5	6.1	5.5	4.4	4.2
Q	Gln	3.9	0.3	4.7	5.2	4.0	4.3
R	Arg	5.8	0.6	5.7	5.5	4.4	5.5
S	Ser	7.2	0.5	8.1	8.3	8.9	6.4
T	Thr	5.3	0.2	5.3	5.7	5.9	5.7
V	Val	6.2	0.5	6.1	5.9	5.6	7.0
W	Typ	1.2	0.1	1.3	1.0	1.0	1.4
Y	Tyr	3.2	0.3	2.8	2.9	3.4	3.0

DM, *Drosophila melanogaster*; EC, *Escherichia coli*; HS, *Homo sapiens*; SC, *Saccharomyces cerevisiae*.

**Table 4:** Codon usage of translations by nematode species

			Species (codons [*n*])															
AA		Codon	*NA *(192,756)	*AC *(305,036)	*AY *(387,372)	*NB *(75,934)	*HC *(584,513)	*OO *(222,616)	*TD *(194,351)	*CE *(9,784,215)	*CB *(8,007,053)	*PP *(297,605)	*SS *(367,308)	*SR *(320,875)	*PT *(284,785)	*PE *(45,802)	*GP *(65,699)	*GR *(290,614)
			
A	Ala	GCA	28.8	25.0	23.9	19.7	26.0	24.9	25.4	31.5	26.1	20.7	33.2	31.4	24.2	31.4	20.4	22.1
A	Ala	GCC	18.5	23.0	24.2	30.0	22.8	23.4	24.0	19.9	22.7	32.4	9.1	9.7	23.4	29.5	31.9	32.6
A	Ala	GCG	16.0	17.7	17.3	17.6	13.6	16.3	16.6	13.1	15.2	16.7	2.0	1.6	7.1	13.2	26.7	27.3
A	Ala	GCT	36.7	34.2	34.6	32.7	37.6	35.4	34.0	35.5	35.9	30.2	55.7	57.4	45.3	25.9	20.9	18.1
Codons per AA			13,237	21,208	29,600	5,916	43,104	16,126	14,513	618,499	502,187	22,118	20,285	17,201	17,930	3,173	4,494	21,522
C	Cys	TGT	54.0	46.9	44.4	39.5	46.7	49.0	44.3	55.3	56.9	39.2	84.4	85.3	63.9	42.6	42.1	43.7
C	Cys	TGC	46.0	53.1	55.6	60.5	53.3	51.0	55.7	44.7	43.1	60.8	15.6	14.7	36.1	57.4	57.9	56.3
Codons per AA			4,538	7,295	8,510	1,702	13,213	5,109	5,154	196,660	159,737	5,789	6,996	6,285	5,682	925	1,584	6,219

D	Asp	GAC	40.5	46.8	47.4	48.5	40.8	45.1	45.4	32.4	35.6	43.0	13.5	12.7	29.1	39.1	61.8	63.8
D	Asp	GAT	59.5	53.2	52.6	51.5	59.2	54.9	54.6	67.6	64.4	57.0	86.5	87.3	70.9	60.9	38.2	36.2
Codons per AA			9,934	15,229	21,124	4,117	32,318	11,797	10,014	520,465	423,125	16,048	20,825	17476	15,182	2,448	2,930	14,122

E	Glu	GAA	58.8	52.0	49.5	46.3	57.9	56.8	55.3	62.5	59.1	37.7	80.4	84.6	65.3	60.4	50.2	49.7
E	Glu	GAG	41.2	48.0	50.5	53.7	42.1	43.2	44.7	37.5	40.9	62.3	19.6	15.4	34.7	39.6	49.8	50.3
Codons per AA			11,865	18,355	25,474	4,774	38,175	14,008	11,873	638,649	543,774	20,537	25,838	20,812	18,057	3,075	3,570	17,574

F	Phe	TTC	56.4	61.0	67.7	72.3	63.7	63.5	63.9	50.5	58.6	81.9	17.8	17.7	41.5	52.8	43.3	50.0
F	Phe	TTT	43.6	39.0	32.3	27.7	36.3	36.5	36.1	49.5	41.4	18.1	82.2	82.3	58.5	47.2	56.7	50.0
Codons per AA			8,977	14,376	16,102	3,031	24,881	9,726	8,311	464,354	373,697	12,020	15,752	15,138	13,032	1,966	3,650	14,036

G	Gly	GGA	42.7	39.5	39.7	41.4	40.4	40.0	40.9	58.7	60.7	55.6	50.1	51.2	52.3	35.1	26.9	24.8
G	Gly	GGC	17.8	23.3	23.5	24.1	20.9	22.5	22.5	12.5	12.1	20.6	4.3	3.1	10.5	29.7	34.9	41.8
G	Gly	GGG	10.1	10.0	9.5	5.9	9.7	8.8	8.4	8.3	8.4	6.9	5.9	3.9	6.8	11.7	20.6	17.5
G	Gly	GGT	29.4	27.2	27.3	28.6	29.0	28.8	28.2	20.5	18.8	16.9	39.8	41.8	30.4	23.5	17.6	15.9
Codons per AA			12,228	18,073	25,292	5,264	38,407	14,914	13,068	524,163	433,832	19,759	22,207	18,910	18,057	3,189	4,741	18,802

H	His	CAC	43.1	48.8	51.9	55.5	43.5	46.8	45.9	39.5	39.7	53.5	15.7	16.2	35.7	37.2	50.5	52.6
H	His	CAT	56.9	51.2	48.1	44.5	56.5	53.2	54.1	60.5	60.3	46.5	84.3	83.8	64.3	62.8	49.5	47.4
Codons per AA			4,853	8,224	9,726	1,835	14,460	5,779	4,965	226,949	183,283	7,363	7,664	6,679	6,789	1,086	1,589	7,500

I	Ile	ATA	21.9	20.1	16.9	14.4	19.6	19.5	18.5	15.6	13.4	10.5	30.2	30.3	24.3	16.0	15.0	11.5
I	Ile	ATC	35.1	41.2	46.0	49.6	39.9	41.4	42.2	31.2	39.2	57.4	9.1	9.2	27.0	33.1	36.9	37.1
I	Ile	ATT	43.0	38.7	37.2	36.0	40.5	39.2	39.3	53.3	47.4	32.2	60.7	60.6	48.7	50.9	48.1	51.4
Codons per AA			10,621	17,171	20,026	3,808	31,585	11,807	9,787	596,151	477,819	15,856	26,077	23,850	18,935	2,659	3,216	15,144

K	Lys	AAA	50.2	44.1	39.5	36.1	47.5	48.5	45.0	59.3	57.9	24.2	80.4	82.4	56.7	55.5	58.9	53.3
K	Lys	AAG	49.8	55.9	60.5	63.9	52.5	51.5	55.0	40.7	42.1	75.8	19.6	17.6	43.3	44.5	41.1	46.7
Codons per AA			12,606	19,080	24,922	5,451	41,187	14,926	12,874	622,428	511,710	19,693	29,246	25,932	23,023	3,465	4,650	17,224

L	Leu	CTA	11.8	10.7	9.7	9.0	10.2	10.9	9.8	9.2	9.9	7.3	6.2	5.5	5.5	7.4	4.4	3.6
L	Leu	CTC	17.2	19.5	21.1	22.8	18.6	20.0	20.7	17.3	18.8	36.9	3.4	3.5	15.2	15.0	18.4	16.0
L	Leu	CTG	16.1	21.6	23.5	24.6	18.8	19.5	20.5	14.1	16.0	20.1	1.7	1.5	9.3	17.4	21.8	25.2
L	Leu	CTT	23.1	19.6	20.2	18.9	22.7	21.2	21.8	24.6	21.1	18.4	30.9	30.5	24.8	20.6	19.3	17.4
L	Leu	TTA	12.5	9.6	7.2	6.2	9.9	8.8	7.7	11.4	9.0	4.8	45.8	46.6	29.4	11.3	8.6	6.1
L	Leu	TTG	19.3	19.0	18.3	18.4	19.9	19.7	19.5	23.3	25.2	12.5	12.0	12.3	15.8	28.3	27.5	31.7
Codons per AA			16,664	27,074	32,761	6,176	49,075	18,693	16,399	841,631	680,113	25,061	30,422	27,556	23,764	3,828	6,003	27,096

M	Met	ATG	100.0	100.0	100.0	100.0	100.0	100.0	100.0	100.0	100.0	100.0	100.0	100.0	100.0	100.0	100.0	100.0
Codons per AA			5,102	8,525	9,696	1,943	14,531	5,794	5,165	255,677	209,897	7,598	8,490	7,569	6,725	1,189	1,489	7,131

N	Asn	AAC	48.0	52.3	53.9	58.0	46.9	48.9	50.7	37.8	42.2	49.7	13.3	13.7	31.5	35.9	53.0	53.7
N	Asn	AAT	52.0	47.7	46.1	42.0	53.1	51.1	49.3	62.2	57.8	50.3	86.7	86.3	68.5	64.1	47.0	46.3
Codons per AA			8,173	12,784	15,647	3,003	24,165	9,013	8,111	477,965	383,675	11,638	21,928	19,815	15,025	2,073	2,589	12,490

P	Pro	CCA	35.0	35.0	33.6	31.2	34.6	35.7	35.1	52.8	53.1	15.3	66.2	66.5	48.2	44.0	20.0	22.5
P	Pro	CCC	14.0	15.9	16.5	16.7	15.8	15.0	14.5	9.1	10.3	38.3	3.7	3.2	17.3	16.2	28.1	23.4
P	Pro	CCG	20.0	21.8	23.1	28.4	19.5	20.9	22.0	19.8	19.1	17.7	2.4	2.1	8.5	19.9	33.0	39.1
P	Pro	CCT	31.0	27.3	26.8	23.7	30.0	28.5	28.4	18.2	17.5	28.6	27.7	28.2	26.0	19.9	18.9	15.1
Codons per AA			9,552	14,020	19,732	3,711	28,449	11,512	9,972	481,470	403,504	15,120	16,634	13,870	12,379	2,068	3,838	14,519

Q	Gln	CAA	56.3	48.9	45.4	42.6	52.6	52.7	52.3	65.6	63.3	37.3	89.1	88.4	69.6	62.9	52.8	52.7
Q	Gln	CAG	43.7	51.1	54.6	57.4	47.4	47.3	47.7	34.4	36.7	62.7	10.9	11.6	30.4	37.1	47.2	47.3
Codons per AA			7,217	11,341	14,843	2,691	21,339	8,424	7,339	405,452	332,326	10,116	13,651	11,696	10,098	2,058	2,515	12,900

R	Arg	AGA	20.8	18.7	17.1	19.3	18.6	18.4	19.8	29.4	31.8	30.6	50.6	52.2	39.1	13.9	12.8	10.7
R	Arg	CGA	23.0	21.9	21.5	20.7	21.3	23.2	21.4	22.9	22.0	16.4	6.8	6.3	8.3	20.4	14.3	17.9
R	Arg	AGG	11.7	13.3	13.8	10.5	12.5	12.0	12.4	7.4	8.1	9.8	9.8	6.9	9.3	10.2	10.3	8.9
R	Arg	CGC	13.9	16.3	17.3	18.2	14.0	15.0	14.7	9.9	10.0	16.2	3.3	3.5	12.3	19.9	26.3	25.1
R	Arg	CGG	8.0	9.1	8.9	7.9	8.5	9.0	8.4	8.9	8.3	5.9	1.7	1.3	3.5	10.9	17.2	17.3
R	Arg	CGT	22.6	20.7	21.3	23.5	25.0	22.4	23.2	21.6	19.8	21.1	27.8	29.8	27.5	24.7	19.1	20.2
Codons per AA			11,856	19,042	23,683	4,857	35,121	13,588	11,363	511,021	432,791	19,008	17,062	13,744	14,051	2,840	4,378	18,826

S	Ser	AGC	14.0	16.4	16.6	18.0	14.6	14.7	16.2	10.3	9.8	9.8	3.1	2.6	7.7	15.5	17.8	17.5
S	Ser	TCA	21.3	19.6	18.1	16.4	21.1	21.4	19.7	25.5	20.4	14.8	36.6	37.6	27.8	24.1	13.5	13.8
S	Ser	TCC	13.6	15.6	16.2	16.3	14.0	14.6	14.7	13.2	16.0	20.7	5.2	4.1	13.2	16.9	20.7	21.0
S	Ser	AGT	14.8	13.4	12.6	11.8	14.3	13.4	14.0	15.0	14.4	10.4	23.8	21.7	17.5	12.9	11.6	11.9
S	Ser	TCG	16.6	16.8	18.6	22.2	16.9	18.2	17.6	15.1	16.7	21.4	2.2	2.0	8.0	14.3	20.3	22.7
S	Ser	TCT	19.7	18.1	18.0	15.5	19.1	17.7	17.8	20.8	22.7	22.9	29.2	31.9	25.8	16.1	16.1	13.1
Codons per AA			13,892	22,627	27,519	5,278	37,542	15,230	13,768	787,872	641,565	22,591	26,438	22,992	19,598	2,921	4,637	21,063

T	Thr	ACA	30.6	28.0	24.8	21.6	27.1	28.4	28.3	34.2	29.6	16.3	51.5	50.9	38.6	35.0	21.9	22.6
T	Thr	ACC	20.4	22.9	25.5	30.8	22.8	23.2	22.4	17.9	21.8	25.7	7.6	7.5	20.8	22.9	27.4	26.8
T	Thr	ACG	18.9	21.8	22.6	22.6	19.9	21.5	22.1	15.2	17.2	24.9	3.9	3.2	9.2	16.3	27.9	28.8
T	Thr	ACT	30.1	27.3	27.2	25.0	30.2	26.9	27.2	32.7	31.4	33.2	36.9	38.3	31.4	25.7	22.7	21.7
Codons per AA			10,197	16,333	20,529	3,959	30,547	11,909	10,364	571,606	461,093	15,952	20,070	17,360	15,532	2,255	3,164	14,970

V	Val	GTA	18.7	16.9	14.6	12.9	18.1	16.2	15.8	15.9	13.7	11.9	26.5	26.5	19.0	12.8	8.3	7.3
V	Val	GTC	21.2	24.7	25.1	28.6	24.9	24.3	25.4	21.8	26.3	34.1	10.0	8.5	20.3	24.5	26.3	28.2
V	Val	GTG	26.8	29.3	30.8	31.8	26.3	29.5	29.1	23.4	25.5	30.6	5.1	3.8	15.8	28.0	37.3	39.2
V	Val	GTT	33.2	29.1	29.5	26.7	30.8	30.0	29.7	38.9	34.6	23.5	58.4	61.2	44.9	34.7	28.1	25.3
Codons per AA			12,606	20,139	25,863	5,243	39,506	14,814	12,547	605,528	491,117	18,939	21,158	18,523	17,487	2,707	3,946	18,216

W	Typ	TGG	100.0	100.0	100.0	100.0	100.0	100.0	100.0	100.0	100.0	100.0	100.0	100.0	100.0	100.0	100.0	100.0
Codons per AA			2,289	3,952	4,517	833	7,229	2,494	2,424	107,642	90,785	3,498	3,508	3,090	2,830	531	791	3,496

Y	Tyr	TAC	51.6	56.5	59.8	62.8	52.7	54.6	55.7	44.0	47.1	64.9	18.5	18.0	36.6	39.4	57.1	61.6
Y	Tyr	TAT	48.4	43.5	40.2	37.2	47.3	45.4	44.3	56.0	52.9	35.1	81.5	82.0	63.4	60.6	42.9	38.4
Codons per AA			6,322	10,100	11,774	2,335	19,614	6,915	6,328	307,728	250,436	8,842	12,998	12,250	10,470	1,346	1,894	7,748

			Species (codons [*n*])															
AA		Codon	*HG *(742,990)	*Mi *(366,435)	*Mj *(203,135)	*Ma *(176,816)	*Mh *(407,985)	*Mc *(205,612)	*ZP *(16,723)	*AS *(646,740)	*AL *(42,919)	*TC *(103,065)	*BM *(561,296)	*DI *(126,880)	*OV *(299,336)	*TS *(290,794)	*TM *(147,995)	*TV *(106,071)
			
A	Ala	GCA	22.3	32.2	32.3	32.8	34.9	36.9	19.5	32.7	31.7	29.3	39.1	40.0	37.5	31.7	24.1	26.7
A	Ala	GCC	33.0	13.3	12.8	11.6	11.4	11.2	24.9	18.1	20.9	20.6	12.3	11.7	13.8	16.4	27.7	25.9
A	Ala	GCG	25.9	8.1	8.0	7.0	7.3	6.8	7.4	22.8	23.9	23.7	12.3	12.8	14.3	18.4	22.7	22.1
A	Ala	GCT	18.7	46.3	46.9	48.6	46.4	45.2	48.2	26.4	23.5	26.4	36.3	35.5	34.4	33.5	25.5	25.3
Codons per AA			54,049	21,050	11,150	10,069	22,749	10,844	1,264	45,388	3,142	7,531	31,439	7,087	17,813	18,017	10,515	7,439.0

C	Cys	TGT	44.5	71.4	72.8	73.7	74.3	72.9	56.3	48.8	48.8	42.8	61.2	62.9	60.0	51.8	31.8	33.8
C	Cys	TGC	55.5	28.6	27.2	26.3	25.7	27.1	43.8	51.2	51.3	57.2	38.8	37.1	40.0	48.2	68.2	66.2
Codons per AA			16,227	7,328	4,217	3,502	8,174	4,656	256	16,446	1,120	2,901	14,026	2,998	6,689	7,559	4,428	3,174.0

D	Asp	GAC	65.5	26.5	27.1	24.8	24.2	22.2	32.1	35.2	41.0	40.5	24.0	20.5	20.6	33.2	56.3	56.1
D	Asp	GAT	34.5	73.5	72.9	75.2	75.8	77.8	67.9	64.8	59.0	59.5	76.0	79.5	79.4	66.8	43.7	43.9
Codons per AA			36,495	17,627	9,747	8,876	19,863	9,894	873	31,446	1,970	5,114	26,014	6,223	15,056	14,599	7,378	5,214.0

E	Glu	GAA	55.7	75.3	74.6	76.2	76.6	78.4	77.9	56.5	55.4	52.8	73.9	77.0	77.1	79.0	61.4	61.8
E	Glu	GAG	44.3	24.7	25.4	23.8	23.4	21.6	22.1	43.5	44.6	47.2	26.1	23.0	22.9	21.0	38.6	38.2
Codons per AA			45,714	24,640	13,289	12,192	27,589	13,117	1,041	39,335	2,469	6,214	31,136	7,577	18,333	18,001	8,907	6,459.0

F	Phe	TTC	48.9	24.3	21.8	20.9	21.3	18.1	55.3	54.5	56.6	60.0	35.0	37.3	36.9	34.3	53.0	52.6
F	Phe	TTT	51.1	75.7	78.2	79.1	78.7	81.9	44.7	45.5	43.4	40.0	65.0	62.7	63.1	65.7	47.0	47.4
Codons per AA			37,855	18,687	11,103	9,412	21,612	11,345	704	29,754	2,102	4,296	30,333	6,568	14,714	14,907	6,593	4,737.0

G	Gly	GGA	25.5	44.5	44.7	45.8	45.8	46.2	33.3	31.2	31.0	32.5	32.7	35.4	35.0	31.1	29.9	31.7
G	Gly	GGC	41.8	14.8	14.9	13.7	14.0	13.0	19.2	25.0	28.1	26.4	16.8	16.9	17.2	24.6	33.8	33.2
G	Gly	GGG	15.7	13.7	13.6	13.0	10.0	8.6	4.9	10.6	11.1	10.6	10.2	7.9	9.2	9.1	9.5	11.0
G	Gly	GGT	17.0	27.1	26.7	27.5	30.2	32.1	42.6	33.2	29.8	30.5	40.2	39.8	38.6	35.3	26.9	24.0
Codons per AA			46,667	20,413	10,992	10,064	22,693	11,194	1,186	37,251	2,633	7,031	27,963	5,944	16,849	14,863	9,577	6,196.0

H	His	CAC	51.6	28.1	27.8	24.8	25.4	22.3	33.7	38.8	41.3	43.8	29.2	22.2	25.2	39.0	51.0	49.7
H	His	CAT	48.4	71.9	72.2	75.2	74.6	77.7	66.3	61.2	58.7	56.2	70.8	77.8	74.8	61.0	49.0	50.3
Codons per AA			19,477	7,978	4,459	3,819	9,003	4,628	421	16,467	1,060	2,710	14,787	3,389	7,427	7,245	3,626	2,601.0

I	Ile	ATA	9.7	23.0	23.1	22.9	23.5	25.3	13.8	23.2	21.9	19.6	29.5	29.2	27.5	25.0	26.6	27.0
I	Ile	ATC	35.7	12.5	11.7	11.2	11.0	9.8	36.5	36.2	39.7	40.7	22.3	23.0	24.7	21.5	29.7	27.9
I	Ile	ATT	54.7	64.5	65.1	65.9	65.5	64.8	49.7	40.6	38.3	39.7	48.3	47.8	47.7	53.5	43.6	45.2
Codons per AA			38,860	23,849	13,986	12,183	28,528	15,200	1,018	38,551	2,582	5,486	39,971	9,443	20,692	17,746	7,330	5,428.0

K	Lys	AAA	60.2	72.5	72.6	73.2	74.7	76.6	61.2	53.8	53.4	53.4	69.0	69.4	69.5	69.4	44.6	47.3
K	Lys	AAG	39.8	27.5	27.4	26.8	25.3	23.4	38.8	46.2	46.6	46.6	31.0	30.6	30.5	30.6	55.4	52.7
Codons per AA			44,829	26,575	15,780	12,963	29,876	15,288	1,324	40,639	2,742	7,563	37,793	8,913	21,167	19,233	9,334	6,794.0

L	Leu	CTA	3.1	6.7	6.4	6.3	6.3	6.9	8.1	10.4	9.9	10.5	10.3	9.9	9.5	5.8	7.9	8.7
L	Leu	CTC	17.1	7.1	6.6	6.0	6.4	5.7	15.9	18.9	19.3	19.1	7.8	7.2	7.6	6.9	10.9	11.1
L	Leu	CTG	21.5	5.0	4.7	4.7	4.8	4.5	4.5	14.8	16.1	18.0	13.0	10.9	13.8	16.0	24.7	23.4
L	Leu	CTT	17.9	24.7	25.0	25.2	25.3	26.0	25.1	21.2	20.7	20.5	19.7	19.8	19.6	17.3	18.0	18.6
L	Leu	TTA	6.6	30.9	31.4	32.3	33.4	35.3	17.5	13.5	12.8	10.1	26.1	27.8	24.6	17.3	9.2	9.7
L	Leu	TTG	33.8	25.6	25.9	25.5	23.8	21.7	29.0	21.3	21.2	21.7	23.1	24.5	24.9	36.8	29.4	28.5
Codons per AA			68,607	34,549	19,485	17,118	38,589	20,003	1,397	56,369	3,564	8,233	53,591	12,048	26,576	27,491	13,110	9,558

M	Met	ATG	100.0	100.0	100.0	100.0	100.0	100.0	100.0	100.0	100.0	100.0	100.0	100.0	100.0	100.0	100.0	100.0
Codons per AA			18,014	8,593	4,930	4,095	9,532	4,730	306	16,510	1,091	2,477	15,883	3,373	7,990	7,625	3,836	2,785

N	Asn	AAC	48.6	22.3	20.9	19.9	18.8	17.6	46.6	43.2	44.1	49.1	27.2	23.8	25.1	34.9	53.1	53.1
N	Asn	AAT	51.4	77.7	79.1	80.1	81.2	82.4	53.4	56.8	55.9	50.9	72.8	76.2	74.9	65.1	46.9	46.9
Codons per AA			33,133	20,562	11,306	9,756	23,509	12,351	779	28,917	1,836	4,201	28,196	6,554	14,572	14,343	5,930	4,424

P	Pro	CCA	24.2	40.6	39.9	41.3	44.8	45.0	46.5	36.9	32.5	34.9	45.0	45.5	44.2	38.3	30.0	30.3
P	Pro	CCC	24.5	10.6	9.6	8.8	7.9	7.2	14.2	14.9	18.4	17.1	10.7	8.5	9.7	10.9	17.7	17.1
P	Pro	CCG	34.9	10.5	11.2	10.7	8.5	8.5	11.7	24.7	24.5	24.6	19.1	19.2	22.9	24.4	28.4	28.7
P	Pro	CCT	16.4	38.3	39.3	39.1	38.9	39.3	27.6	23.5	24.7	23.4	25.2	26.8	23.2	26.3	23.8	23.9
Codons per AA			36,929	16,614	8,537	7,305	17,440	8,307	768	30,784	2,276	5,519	23,108	4,915	13,464	11,909	7,466	5,066

Q	Gln	CAA	57.1	79.4	79.6	80.2	80.9	80.3	82.0	57.4	57.1	50.2	61.4	63.6	62.8	59.9	50.4	50.3
Q	Gln	CAG	42.9	20.6	20.4	19.8	19.1	19.7	18.0	42.6	42.9	49.8	38.6	36.4	37.2	40.1	49.6	49.7
Codons per AA			33,107	16,926	8,552	7,532	17,739	8,805	640	23,271	1,514	3,612	21,428	4,769	11,797	11,420	5,644	4,047

R	Arg	AGA	11.6	28.8	28.1	29.2	30.0	30.0	16.4	17.7	16.3	17.2	22.3	21.9	21.2	21.8	16.3	17.6
R	Arg	CGA	18.0	16.8	16.8	17.3	16.7	17.2	17.3	21.3	18.5	19.7	24.0	26.3	25.4	22.7	18.1	17.7
R	Arg	AGG	9.0	9.7	9.4	8.8	8.9	8.6	2.9	12.3	11.8	12.4	10.9	8.8	9.5	7.1	12.4	13.2
R	Arg	CGC	25.0	9.4	9.2	8.5	8.8	7.9	15.3	15.8	20.2	17.3	9.6	9.4	10.4	13.7	22.9	21.8
R	Arg	CGG	15.8	5.6	5.4	4.9	4.5	4.5	1.2	8.3	7.9	7.2	9.2	8.4	8.8	8.5	11.6	11.5
R	Arg	CGT	20.6	29.7	31.0	31.4	31.1	31.8	47.0	24.6	25.3	26.2	24.0	25.2	24.8	26.1	18.7	18.2
Codons per AA			47,178	19,486	11,042	9,171	21,409	9,973	1,001	40,766	2,650	6,429	31,420	7,457	17,634	16,384	9,203	6,468

S	Ser	AGC	15.5	9.1	9.4	8.4	7.9	7.6	11.8	16.4	16.6	17.1	11.0	9.7	11.1	15.1	23.2	22.5
S	Ser	TCA	14.4	24.5	24.3	24.1	27.0	28.6	18.1	22.3	20.8	19.6	28.4	29.5	27.7	21.0	14.4	15.5
S	Ser	TCC	20.8	9.0	8.6	7.9	6.8	6.6	15.7	9.9	12.0	11.0	10.4	9.3	10.7	10.3	17.7	17.3
S	Ser	AGT	12.5	17.6	17.2	18.5	18.1	18.4	13.6	15.8	13.8	14.4	19.2	18.7	19.1	19.1	13.3	13.8
S	Ser	TCG	22.4	7.8	8.3	7.9	6.2	6.2	13.3	21.4	22.3	24.8	12.2	12.8	14.0	14.4	19.1	18.3
S	Ser	TCT	14.3	31.9	32.1	33.3	34.0	32.5	27.5	14.3	14.4	13.1	18.9	20.0	17.5	20.1	12.2	12.6
Codons per AA			54,466	27,606	14,239	12,353	29,702	15,207	899	48,736	3,157	6,860	43,504	9,483	21,762	22,175	10,980	7,907

T	Thr	ACA	23.2	40.6	39.2	41.1	42.3	42.7	19.5	30.2	30.3	27.5	39.0	40.1	38.1	31.8	21.3	25.1
T	Thr	ACC	25.6	11.1	9.9	9.0	9.1	7.9	28.6	19.2	22.4	20.0	15.6	12.9	15.6	16.3	24.9	23.1
T	Thr	ACG	26.6	8.4	8.8	8.6	8.5	7.7	12.5	26.8	24.9	29.2	15.4	16.3	17.4	19.8	30.9	30.3
T	Thr	ACT	24.6	39.8	42.1	41.4	40.1	41.7	39.4	23.8	22.4	23.2	30.0	30.7	28.9	32.1	22.9	21.5
Codons per AA			37,607	19,046	10,254	8,837	20,194	10,213	919	34,207	2,219	5,814	29,901	6,460	15,488	14,901	7,680	5,829

V	Val	GTA	6.0	17.6	16.9	17.5	17.0	20.7	14.1	16.4	16.6	16.4	25.4	26.2	26.3	19.4	16.6	17.7
V	Val	GTC	29.6	13.2	12.3	11.9	11.4	10.7	29.2	20.6	21.7	21.3	14.3	14.0	14.4	16.7	22.8	21.9
V	Val	GTG	38.3	13.5	13.2	11.7	12.9	11.7	12.9	29.7	31.1	30.7	22.9	21.2	21.6	26.3	27.6	28.3
V	Val	GTT	26.1	55.8	57.6	58.9	58.6	56.9	43.8	33.2	30.6	31.5	37.5	38.7	37.7	37.6	33.0	32.1
Codons per AA			45,942	19,706	10,925	9,899	21,917	10,703	1,109	42,490	2,780	6,580	33,067	7,389	17,037	18,805	9,977	7,169

W	Typ	TGG	100.0	100.0	100.0	100.0	100.0	100.0	100.0	100.0	100.0	100.0	100.0	100.0	100.0	100.0	100.0	100.0
Codons per AA			9,033	4,055	2,294	2,002	4,746	2,298	188	7,902	532	1,213	6,380	1,377	3,584	3,486	1,777	1,350

Y	Tyr	TAC	58.9	23.2	22.2	20.9	21.4	19.6	34.6	37.3	41.2	46.0	30.3	26.0	28.4	38.7	61.8	62.7
Y	Tyr	TAT	41.1	76.8	77.8	79.1	78.6	80.4	65.4	62.7	58.8	54.0	69.7	74.0	71.6	61.3	38.2	37.3
Codons per AA			18,687	11,019	6,656	5,582	12,940	6,716	627	21,299	1,453	3,267	20,353	4,785	10,337	9,939	4,691	3,401

Values are given as % per AA, or as numbers for Codons per AA. Definitions of species two letter codes are provided in Table 1. AA, amino acid.

**Figure 1:**
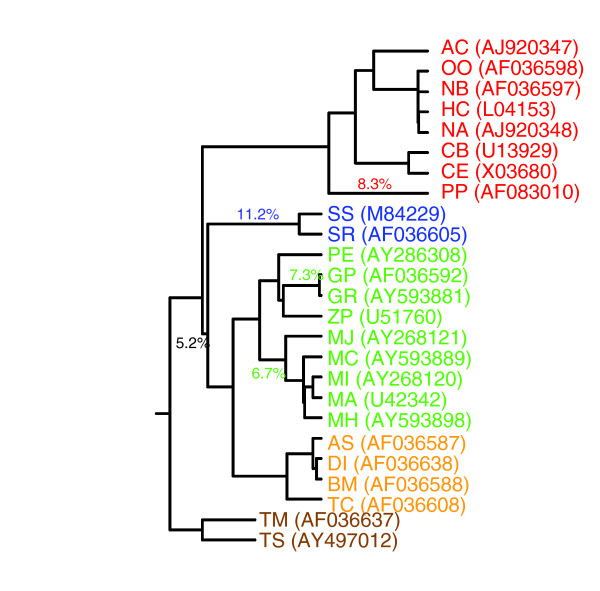
Maximum likelihood (ML) analysis of 18S ribosomal RNA from 25 nematode species. The ML calculation assumes a molecular clock; thus, the tips of the tree are all equidistant, in branch length, from its root. This model of base substitution allows the expected frequencies of the four bases to be unequal, and different rates of evolution at different sites are allowed. The numbers indicate reconstruction of percentage changes in overall codon usage on this phylogenetic topology (see Codon usage patterns and relationships to sampling method, nematode phylogeny, and GC content [under Results]). A distance matrix of D values corrected for non-additivity [1 - antilog(-D)] × 100 was partitioned on the topology using the cyclic neighbor-joining algorithm, as illustrated by Avise [82]. Approximate percentage change in overall codon usage is indicated for five branches inferred to have undergone 5% or more divergence from an ancestral nematode pattern. This analysis identified genera *Globodera*, *Meloidogyne*, *Pristionchus*, and *Strongyloides *as having the most highly derived patterns of codon usage, and the remaining species as having relatively little net divergence from an ancestral nematode pattern. Definitions of species two letter codes are provided in Table 1; GenBank accession numbers are listed on right. Clades V are shown in red, IVa in blue, IVb in green, III in yellow, and I in brown.

To examine these variables independent of species relatedness, correlations were calculated using phylogenetically independent contrasts (see Materials and methods, below). The variances of the contrasts were computed for each character as a measure of the variance accumulating per unit branch length. The branch lengths were estimated from the maximum likelihood phylogeny assuming a molecular clock (Figure 1); by this criterion, the tips of the tree are all equidistant in branch length from its root. Computed contrasts were plotted in all figures representing pair-wise comparisons, and the correlation coefficients were calculated from the paired contrasts. This method is robust to changes in molecular clock assumptions. (Trees calculated without the assumption of a molecular clock are similar in topology but differ in rooting, and branch lengths vary according to amount of base substitution in the 18S rRNA; the clock-based tree provides branch lengths that should estimate most closely the relative durations of branches in evolutionary time. Because independent contrasts are influenced mainly by relative branch lengths, our results should be robust to alternative placements of the root.)

### Amino acid composition of nematode proteins and relationship to GC content

AA composition of predicted proteins in nematodes varies among species within a narrow window and is similar to that observed in other organisms (Tables 2 and 3). (Standard deviations in AA usage among nematodes range from 5% to 15% of mean usage, and mean nematode AA usage differs from the mean of four representative organisms by an average of 8%.) Across nematodes, Leu is the most common AA (8.8% of all codons) and Trp the least common (1.1%). Eight AAs contribute an average of more than 6% each to AA content (Ile, Gly, Val, Glu, Ala, Lys, Ser, and Leu); these AAs are also among the most common in the proteomes of other representative species, including humans (Table 3). As in other taxa [46], nematodes show a correlation between AA usage and the degree of codon degeneracy (R = 0.72).

**Figure 2:**
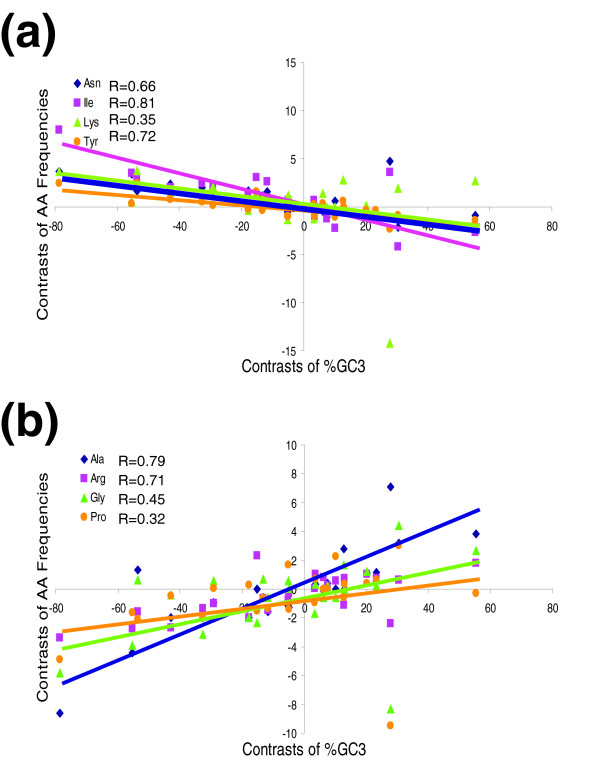
Correlation between phylogenetically independent contrasts of coding sequence GC3 content and AA usage for 25 nematode species. **(a) **AAs lysine (Lys), isoleucine (Ile), asparagine (Asn), and tyrosine (Tyr) are used less frequently as the species' coding sequence GC3 content increases. **(b) **AAs alanine (Ala), glycine (Gly), arginine (Arg), and proline (Pro) are used more frequently as the coding sequence GC3 content increases. AA, amino acid.

In nematodes, coding sequence GC content, derived from our EST clusters, varies from 32% to 51% (Table 1) among species, with a mean of 43.6 ± 5.9%. The distribution is biphasic, with a peak at 36% GC and a second peak at 48%. *Strongyloides *(*SS *and *SR*), *Meloidogyne *(*MI*, *MJ*, and so on), and filarial parasites (*BM*, *DI*, and *OV*) are the most AT rich (low GC); and *NB*, *PP*, and cyst nematodes (*GP*, *GR*, and *HG*) are the most GC rich (approximately 50%). The variation observed in AA composition among species shows a clear relationship to the species' coding sequence GC content. The frequency of AAs encoded by WWN codons (AA, AT, TA, or TT in the first and second nucleotide positions; Asn, Ile, Lys, Try, Phe, and Met) decreases with increasing coding sequence GC content (Figure 2a), whereas the proportion of AAs encoded by SSN codons (GG, GC, CG, and CC; Ala, Arg, Pro, and Gly) increases with higher coding sequence GC content (Figure 2b), and these relationships remain even after removing the effect of evolutionary relationships using phylogenetically independent contrasts. Among AAs, the most uniform and precipitous decrease with increasing GC content was seen with Ile and Tyr whereas the most uniform and rapid increase with higher GC content was seen with Ala and Arg. The trend is less pronounced for other AAs (flatter slope, lower R value). Thr, encoded by four GC/AT 'balanced' codons (ACN), exhibits no change in its frequency with changing GC content (data not shown).

### Base composition by codon position in nematode transcripts and relationship to GC content

Codon usage in nematode species was examined by several methods, including comparison of base usage by position (1-3) over all AAs and comparison of codon usage within each AA. Over all AAs, purine (AG) and pyrimidine (TC) usage in positions 1, 2, and 3 is remarkably uniform between species, favoring purines in position 1 (AG 59.6 ± 1.5%), near equal usage in position 2 (AG 50.0 ± 0.8%), and pyrimidines in position 3 (AG 47.9 ± 1.5%; Additional data file 1). Similar values were observed in *Schistosoma mansoni *(AG 61%, 53%, and 48% in positions 1, 2, and 3, respectively) [1]. GC versus AT usage also varies by position but with much greater variance, with near equal usage in position 1 (50.3% GC) and lower GC usage in positions 2 and 3 (39.1 and 41.4%, respectively), mainly due to greater use of G in position 1 and T in positions 2 and 3 [4].

**Figure 3:**
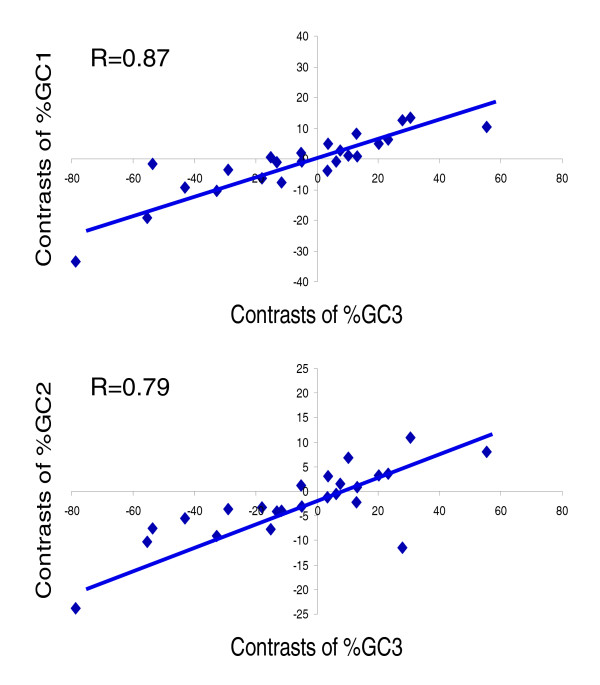
Correlation between phylogenetically independent contrasts of the third position GC content (GC3) and that of the first (GC1) and second (GC2) codon positions for 25 nematode species.

The variation observed in GC usage by codon position among species exhibits a clear relationship to the species' overall coding sequence GC content. Not surprisingly, both GC1 and GC2 composition increase with higher coding sequence GC3 content (Figure 3). Specifically, species with high AT content like root-knot *Meloidogyne *species (*MI*, *MJ*, and so on) and filarial worms (*BM*, *DI*, and *OV*) [38,39] are biased toward codons terminating in A or T, whereas species with higher GC content such as *NB*, *PP*, cyst nematodes, and whipworms (*TM *and *TV*) prefer codons ending with G or C. Differences in calculated GC composition by codon position (1-3) between species are determined both by the species' AA usage (as described above) and the codons used for each AA. For example, Cys was encoded by TGT as much as 85% of the time for the AT-rich *Strongyloides *genomes, whereas TGC was used up to 60% of the time in GC-rich genomes such as *NB*, *PP*, and *HG*. To compare codon usage more systematically for individual AAs between species, we employed a statistical approach (described in Materials and Methods and in the following section).

### Codon usage patterns and relationships to sampling method, nematode phylogeny, and GC content

Similarity in codon usage was quantified and reported as D_100 _values for each species and AA compared [47,48] (matrix of D_100 _values for each species and AA compared is available in Additional data file 2).

Because analyses of all but two of the nematode species were based on EST-derived partial genomes [26], comparisons were performed to estimate the differences in codon usage pattern that could be expected using EST collections versus gene predictions derived from a fully assembled and annotated genome. Using *C. elegans*, parallel analyses were performed using either all 22,254 predicted gene products or two EST datasets (*CE*-A and *CE*-B) each comprising 10,000 ESTs. Clustering and peptide predictions were performed using the same algorithms as for the other 30 species. The average D_100 _value for the comparison of codon usage pattern between the *CE*-A and *CE*-B datasets was 0.18, which was not statistically different at the *P *< 0.05 threshold and less than the D_100 _value of the *C. elegans *to *C. briggsae *comparison (0.40). Comparing the *CE*-A and *CE*-B datasets to the genome-derived full gene set for *C. elegans *yielded average D_100 _values of 0.67 and 0.26, respectively. At a practical level, the calculated use of the average codon in *C. elegans *based on *CE*-A and *CE*-B differs from that based on prediction from the whole genome by just 3.4 ± 2.3% and 2.0 ± 1.5%, respectively. Therefore, although differences in calculated codon usage using partial versus whole genome data are modest enough to make EST-derived codon usage data highly informative, care must be taken not to over-interpret minor differences in D_100 _values because such differences are probably within the range of sampling error (see Discussion, below). However, such uncertainty around small differences in D_100 _values does not alter the major trends that we describe.

The 16 intragenus comparisons of species sharing the same genus name (*Ancylostoma*, *Caenorhabditis*, *Strongyloides*, *Globodera*, *Meloidogyne*, *Ascaris*, and *Trichuris*) all have low D_100 _values, with a mean of 0.14 ± 0.11 (median 0.09, range 0.02-0.40), indicating very similar patterns of codon usage among species within the same genera. By contrast, the 480 comparisons beyond named genera vary greatly, with a mean D_100 _value of 8.10 ± 7.46 (median 5.26, range 0.08-40.56). Low D_100 _values do sometimes extend to comparisons among genera. For instance, relatively low D_100 _values (0.08-1.94) are observed within the following: order Haemonchidae (*HC*, *OO*, and *TD*); subfamily Heteroderinae (*GP*, *GR*, and *HG*); superfamily Ascaridoidea (*AS*, *AL*, and *TC*); and superfamily Filarioidea (*BM*, *DI*, and *OV*). However, low D_100 _values are not maintained across family Ancylostomatidae (*NA*, *AC*, and *AY*), family Strongyloididae (*SS*, *SR*, and *PT*), superfamily Tylenchoidea (*PE-MC*), and order Trichocephalida (*TS*, *TM*, and *TV*). Similarity in codon usage, as indicated by low D_100 _values, does not extend to the level of the major clades (I, III, IVb, IVa, and V).

Furthermore, species with very similar GC content, although distantly related, can exhibit extremely similar codon usage (for instance *Ancylostoma caninum *versus *Toxocara canis*, GC = 48%, D_100 _= 0.79). Species with the lowest average D_100 _values in one-versus-all comparisons are those closest to the median species GC content, such as *PE *(GC = 46%). Taxa with the highest AT content, such as *Strongyloides *and *Meloidogyne *species, have among the most extreme differences in codon usage when compared with species beyond their genus (median D_100 _values are 15.3 and 9.4, respectively).

Phylogenetic analysis of changes in codon usage using (1 - antilog [-D]) × 100, interpretable as percentage divergence in overall codon usage (Figure 1), identifies five branches that have accumulated more than 5% change in codon usage. These branches are as follows: the most recent common ancestor of clades III, IVa, and IVb (5.2%); the most recent common ancestor of clade IVa (11.2%); the most recent common ancestor of genus *Meloidogyne *(6.7%); the most recent common ancestor of genus *Globodera *(7.3%); and the lineage represented by *PP *(8.3%). Genera *Globodera*, *Meloidogyne*, *Pristionchus*, and *Strongyloides *therefore represent the most highly derived patterns of codon usage in nematodes, with the remaining species exhibiting less relatively divergence from an ancestral nematode pattern.

### Codon bias in nematode transcripts and relationship to GC content

**Figure 4:**
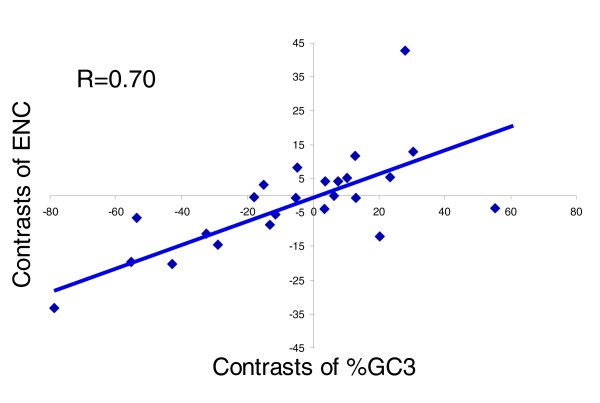
Correlation between phylogenetically independent contrasts of each species' %GC3 and its mean ENC for 25 nematode species. ENC, effective number of codons.

We used the effective number of codons (ENC) to measure the degree of codon bias for a gene [49]. ENC is a general measure of non-uniformity of codon usage and ranges from 20 if only one codon is used for each AA to 61 if all synonymous codons are used equally. The mean ENC across all sampled nematode species is 46.7 ± 5.1, and many nematodes have ENC values similar to those obtained for various bacteria, yeast, and *Drosophila *species (ENCs of 45-48) [50]. Outliers with low ENC values include *SS *and *SR*, for which transcripts on average utilize only about 35 of 61 available codons. The variation observed in ENC values among species exhibits a clear relationship to the species' overall coding sequence GC3 content (R = 0.70 following phylogenetic correction; Figure 4). The correlation confirms that species with lower GC3 content in coding sequence have greater codon usage bias than those with higher GC3. ENC values for nematodes peak at 47-49% GC (data not shown). In addition to comparing species' mean ENC values, we also examined the distribution of ENC values across all transcripts within each species. Although all species have examples of transcripts across nearly the full range of possible ENC values, in species with low GC3 content, such as *SR*, the distribution is shifted toward a lower ENC peak (Additional data file 3).

To ensure that differences in our available data for each species (for instance, cluster number and cluster length) were not creating artifacts in ENC values, quality checks were performed. Unlike measures such as codon bias index, scaled ×2, and intrinsic codon bias index, ENC values should be independent of translated polypeptide length and sample size [49,51], and our analysis confirmed this. No correlation with ENC was observed with either average translated polypeptide length or number of clusters for a species. In fact, *SS *and *SR *with the lowest ENC values had above average cluster length and number. As additional confirmation, we randomly selected 2,400 *C. elegans *genes (the average number of clusters for species other than *CE *and *CB*) and calculated ENC based on either full-length genes or genes trimmed to 121 AAs (the average length cluster translation for species other than *CE *and *CB*). Differences in the average ENC numbers for these datasets were not statistically significantly different from zero (*P *> 0.05).

In addition to codon bias, neighboring nucleotides influence the codon observed at a position relative to synonymous codons. The most important nucleotide determining such context dependent codon bias [52-54] is the first one following the codon (N1 context) [55,56]. An analysis using the complete genesets of *Homo sapiens*, *Drosophila melanogaster*, *C. elegans*, and *Arabidopsis thaliana *revealed that 90% of codons have a statistically significant N1 context-dependent codon bias [57]. Using the same method we calculated that, for the 30 nematode species represented by EST-derived codon data, an average of 63% of codons with N1 context have a statistically significant bias (because the R values differed from 1 by more than 3 standard deviations). Fedorov and colleagues [57] showed that their results were not considerably affected by gene sampling. However, for our dataset the calculated *CE*-A and *CE*-B N1 context with statistically significant bias was 75% and 83% of the codons, respectively, as compared with 96% when the complete *C. elegans *gene set was used. Therefore, the extent of significant N1 context-dependent codon bias determined from EST-based codon usage data may change as more complete nematode genomes become available. The complete list of relative abundance of all nematode species with N1 context, R values, and standard deviations are available in Additional data file 4.

### Coding sequence GC content versus total genome GC content

**Figure 5:**
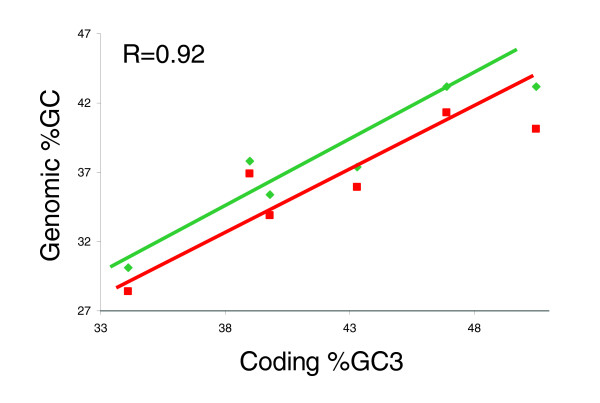
Correlation between coding sequence (transcriptome) %GC3 and genome %GC for six nematode species with extensive available genomic sequence. The green line indicates the coding sequence %GC versus the full genomic %GC. In this case, coding sequence %GC3 is a contributor to the full genomic %GC such that X and Y are not independent variables. The red line indicates the coding sequence %GC3 versus noncoding genomic %GC. In this case, the coding sequence contribution has been removed from genomic totals such that X and Y are independent variables. For *BM*, *TS*, *HC*, and *AC*, the calculation of noncoding genomic %GC relies on the assumption that the species will have a similar breakdown of coding and noncoding sequence as *CE*. Assembly and gene calling for the *BM*, *HC*, *TS*, and *AC *sequences are needed to test this assumption. Definitions of species two letter codes are provided in Table 1.

Because of the clear relationships of AA composition, codon usage pattern, and codon bias to the GC content of coding sequences and the interest in the underlying cause of these correlations (see Discussion, below), we examined the relationship between coding sequence GC3 content and genomic GC content in nematodes. Total genomic GC content was calculated for the six nematode species for which significant genome sequence data were available as unassembled sequences (*TS *and *HC*), partial assemblies (*BM *and *AC*), or finished assemblies (*CE *and *CB*). Noncoding genomic GC content was calculated for *CB *and *CE *based on published estimates of the percentage of each genome that is composed of noncoding sequence, namely 74.5% and 77.1%, respectively [35]. Extrapolations were made for other species using the *CE *percentage noncoding estimate. Although GC content varies across the genome for some organisms (for example, isochors in vertebrates [58]), GC content is fairly uniform across the *C. elegans *genome [17]; furthermore, as yet there is no evidence of non-uniformity in other nematode genomes. A positive correlation was observed between coding GC3 content and both total GC content and extrapolated noncoding GC content (R = 0.92; Figure 5). Noncoding genomic sequences varied across a wider span of GC values than did coding sequences. In all six nematodes, coding sequences were somewhat more GC rich than were noncoding sequences (2-10%).

Comparison of coding sequence GC versus 3'-untranslated region (UTR) GC also supports this conclusion. Calculated 3'-UTR GC for the 30 species in our EST dataset ranges from 28.6% to 46.1%. Correlation between phylogenetically independent contrasts of coding GC content (Table 1) and 3'-UTR has an R value of 0.81 (data not shown).

### Codon usage patterns in abundantly expressed genes and candidate optimal codons

Representation in cDNA library generally correlates with abundance in the original biologic sample [59] although artifacts occur [60,61]. To investigate the difference in the codon usage patterns in highly abundant transcripts as compared with less abundantly expressed genes, as determined by ESTs, we selected five species, each of which is a member of a different clade. The selected species (*AY*, *MI*, *OV*, *SR*, and *TS*) were represented by approximately 3,000 clusters each (range 2,693-3,214), and codon usage tables were generated for subsets of genes from each species: the 20 most abundant clusters versus all remaining clusters, and the 50 most abundant clusters versus all remaining clusters. Results of both comparisons were similar, and for simplicity we discuss only the results based on the comparison of the 50 most abundant versus all remaining clusters. Clusters 51 to about can be described as containing mainly genes with low to moderate expression because transcripts of extremely low abundance are less likely to be represented in EST collections (for instance, neuronal 7-transmembrane receptors). Codon usage tables, AA frequencies, and relative differences between AA usage of the most abundant and less abundant genes are available in Additional data file 5.

D values were calculated across all AAs and the codon usage in each species was generally similar for genes represented by abundant EST clusters and genes represented by low to moderate expression EST clusters. *SR *exhibited the greatest difference between the two usage patterns (D_100 _= 6.15). Additionally, for all the species at least seven AAs were used significantly more frequently in the abundant genes than in the remainder of the genes. For example, although the abundant *OV *clusters had a Pro composition of 10.5% of all AAs, the rest of the clusters were only 4.4% Pro.

Examining the codon usage frequencies within an AA, an increase in usage has been noted with higher gene expression for specific so-called 'optimal' codons [62,63]. Using the codon usage tables for the top 50 and remaining clusters, we have defined a list of potentially optimal codons with usage that is higher in abundant transcripts by a statistically significant measure. Out of the 59 synonymous codons there were 24, 28, 25, 27, and 23 candidate optimal codons (Table 5) in *AY*, *MI*, *OV*, *SR*, and *TS*, respectively. For example, Tyr is encoded by two codons (TAC and TAT); in *AY *TAC is used 75% of the time in the abundant clusters and 59% of the time in the less abundant clusters. Similar analysis documented about 21 candidate optimal codons in *C. elegans *for which usage differed significantly when comparing high and low expressed genes [31,33,64]. Confirmation of these candidate codons as truly 'optimal' will require additional investigations, including other means of verifying relative expression levels (for example, microarrays and reverse transcription [RT]-polymerase chain reaction [PCR]).

**Table 5:** Candidate optimal codons in five species, determined as frequency increase by increased expression level^a^

			Species								Species				
							
AA		Codon	*AY*	*MI*	*OV*	*SR*	*TS*	AA		Codon	*AY*	*MI*	*OV*	*SR*	*TS*
A	Ala	GCA			GCA			N	Asn	AAC	AAC	AAC		AAC	
A	Ala	GCC	GCC	GCC	GCC	GCC		N	Asn	AAT			AAT		
A	Ala	GCT	GCT	GCT		GCT	GCT								
								P	Pro	CCA		CCA	CCA	CCA	CCA
C	Cys	TGC	TGC	TGC	TGC	TGC		P	Pro	CCC	CCC				
								P	Pro	CCG		CCG			
D	Asp	GAC	GAC	GAC	GAC	GAC		P	Pro	CCT					CCT
D	Asp	GAT					GAT								
								Q	Gln	CAA				CAA	CAA
E	Glu	GAA			GAA	GAA	GAA	Q	Gln	CAG	CAG	CAG	CAG		
E	Glu	GAG	GAG	GAG											
								R	Arg	AGA					AGA
F	Phe	TTC	TTC	TTC	TTC	TTC		R	Arg	CGA					CGA
F	Phe	TTT					TTT	R	Arg	AGG			AGG		
								R	Arg	CGC	CGC	CGC		CGC	
G	Gly	GGA	GGA		GGA	GGA	GGA	R	Arg	CGG			CGG		
G	Gly	GGC		GGC				R	Arg	CGT	CGT	CGT		CGT	
G	Gly	GGT	GGT												
								S	Ser	AGC	AGC	AGC	AGC	AGC	AGC
H	His	CAC	CAC	CAC	CAC	CAC	CAC	S	Ser	TCA			TCA		TCA
								S	Ser	TCC	TCC	TCC	TCC	TCC	
I	Ile	ATA					ATA	S	Ser	AGT					AGT
I	Ile	ATC	ATC	ATC	ATC	ATC		S	Ser	TCG	TCG	TCG	TCG		
I	Ile	ATT				ATT		S	Ser	TCT				TCT	
K	Lys	AAA					AAA	T	Thr	ACC	ACC	ACC	ACC	ACC	
K	Lys	AAG	AAG	AAG	AAG	AAG		T	Thr	ACG		ACG	ACG		
								T	Thr	ACT				ACT	ACT
L	Leu	CTA					CTA								
L	Leu	CTC	CTC	CTC		CTC		V	Val	GTA					GTA
L	Leu	CTG			CTG			V	Val	GTC	GTC	GTC	GTC	GTC	
L	Leu	CTT	CTT	CTT		CTT	CTT	V	Val	GTG		GTG			
L	Leu	TTA					TTA	V	Val	GTT		GTT		GTT	
L	Leu	TTG		TTG	TTG	TTG									
								Y	Tyr	TAC	TAC	TAC	TAC	TAC	TAC

^a^Expression level inferred by EST number. Definitions of species two letter codes are provided in Table 1. AA, amino acid; EST, expressed sequence tag.

## Discussion

A comprehensive and well supported codon usage table for 32 nematode species across most of the phylum's major clades and based on nearly 26 million codons is now available. Use of large EST datasets provide an excellent resource for determining a species mean codon usage with results that differ only modestly from those obtained from full genomes. In nematodes, codon usage varies widely, as does coding and noncoding GC content of nematode genomes. GC content correlates with AA usage, similarity of codon usage, and codon bias. Codon usage similarity in Nematoda usually persists within a genus but then rapidly diminishes, even within each major clade (clades I-V). Based on EST sampling, differences in codon usage between highly abundant genes and moderately expressed genes are recognizable, and candidate optimal codons can be identified.

### GC content, causality, and directional mutation pressure

Correlations between GC content and mean codon usage and mean AA usage similar to those we describe across the phylum Nematoda have been observed in many other species [4,65-70]. Directional mutation pressure is a theory proposed to quantify differences in GC content observed in species [3]. Important variables include the relative values of the mutation rates u (GC/CG → AT/TA change) and v (AT/TA → GC/CG change). The preponderance of the evidence supports causality of genome GC content, as determined by directional mutation pressure or nucleotide level selective pressure, driving both codon usage and AA composition rather than the reverse. First, in an examination of sequence data from a large number of a bacteria, archaea, and eukaryotes, a model assuming directional mutation and selection at the nucleotide level with different rates of change for each of the three codon positions can explain 71-87% of the variance in codon usage and 71-79% of the variance in AA composition [5]. Knight and coworkers [5] found that between species an AA's change in frequency in response to GC content is determined by the mean GC content of its codons, whereas a codon's change in frequency is determined by the difference between its GC content and the mean GC content of its synonyms. We observe this result to be generally true across nematodes as well.

Second, an analysis comparing codon usage from eubacterial and archaeal species with complete genomes [6] found that codon usage can be predicted with some accuracy if one knows only the sequence of the species' intergenic sequences from which genome GC content, and context dependent nucleotide bias parameters can be calculated. Using data from six nematode species for which substantial genome sequence data are available, we observed that coding sequence GC3 content correlates with noncoding sequence GC content. This perhaps indicates that, for nematodes too, it should be possible to predict mean codon usage using only knowledge of the intergenic sequences of the species. Our findings are consistent with the model that genome GC content drives both mean codon usage and AA composition.

Little is known about why directional mutation pressure or selective pressure leads to differences in genomic GC content among species [5,6]. Within nematodes we see no pattern based on ecologic niche or other factors that corresponds to GC content. For instance, cyst nematodes (*GP*, *GR*, and *HG*) and root knot nematodes (*MI*, *MJ*, *MA*, *MH*, and *MC*) have similar life cycles as plant sedentary endoparasites, but their coding sequence GC contents are completely different (approximately 50% versus 36%). Whatever the driving forces, it is important for nematologists to note that they are sufficiently strong not only to change base composition in wobble sites (third position) but also to alter first and second codon positions and even AA sequences - features that are sometimes assumed to be under selective pressure for conservation.

### Species' mean codon usage versus optimal codons in abundantly expressed genes

Our use of thousands of genes per species without weighting for abundance of expression has produced a codon usage dataset that probably reflects codon usage for genes with low to moderate abundance of mRNAs. In the case of *C. elegans *and *C. briggsae*, our codon usage table reflects the mean of all predicted genes, although this is similar to that observed based on sampling of 10,000 ESTs. At this 'genome-wide' level, genome GC content is a dominant factor. However, codon usage within a species does vary from gene to gene.

Prior studies of *C. elegans *codon usage have examined codon usage and the role of 'optimal codons' in putatively abundantly expressed genes [31,33,64]. Stenico and coworkers [31] observed differences between usage of specific codons based on 168 known genes, including many highly expressed transcripts (for instance, actin, myosin, collagen, and vitellogenin), and 90 unidentified reading frames (URFs) emerging from sequencing efforts presumed to represent a more 'random sampling' of the genome. Overall, our codon usage results based on the full *C. elegans *genome are similar to both the results from Stenico and coworkers' 168 known genes (D_100 _= 0.97) and the 90 URFs (D_100 _= 1.1). Duret and coworkers [33] weighted 15,425 *C. elegans *genes for expression levels based on their EST abundance and identified 21 favored codons used most frequently in highly expressed genes. In all cases, these optimal codons could be decoded by isoaccepting tRNAs that had the highest gene copy number in the genome, indicating that optimal codons are probably selected for translational efficiency. Likewise, Kanaya and coworkers [64] showed that, in *C. elegans*, ribosomal proteins and histones, selected as representatives of highly expressed genes, also use optimal codons different from those used by average genes and that these optimal codons correspond to tRNA gene copy number. AA frequencies in abundant *C. elegans *genes also correspond to isoaccepting tRNA gene copy number (R^2 ^= 0.67) [33].

Therefore, in *C. elegans *different pictures emerge of evolutionary forces acting on codons and AAs in low to moderately expressed genes (directional mutation pressure, genome GC content) compared with abundantly expressed genes (optimal codons, tRNA copy number). In other nematodes, it is possible that a similar dichotomy exists, although we currently lack knowledge of tRNA gene copy number, and information on gene expression levels is largely limited to estimations based on EST abundance. Here, we have provided candidate optimal codons in *AY*, *MI*, *OV*, *SR*, and *TS*. A more detailed examination of codon usage as it relates to gene expression level in other nematodes will be possible by taking advantage of microarray and RF-PCR confirmation of transcript abundance.

### Implications for phylogenetic studies and molecular biology

The extent to which average nematode genes sequences are susceptible to GC or AT shifts should sound a cautionary note for phylogenetic studies of nematode species, genes, and proteins based solely on coding sequences because convergent evolution may create confusing results. Knight and coworkers [5] noted that, 'Pairs of species with convergent GC contents might also evolve convergent protein sequences, especially at functionally unconstrained positions. For example, the frequencies of both lysine and arginine are highly (but oppositely) correlated with GC content, and lysine and arginine can easily substitute for one another in proteins.' In nematodes as well, one can envision exchanges of Lys and Arg (Figure 2).

For cloning genes of interest from various nematode species, we found that codon usage is a rapidly evolving feature such that codon usage patterns beyond within a genus comparisons are often divergent. Therefore, extrapolating assumed codon usage patterns to unsampled species in nematodes beyond the genus level is unlikely to be successful. At a practical level of species choice, cloning of orthologs and homologs of interest from species that are AT rich and have low ENC values, such as *SS *and *MI *with low ENC values, will require fewer degenerate primers than may be needed for more GC rich species such as *TC *and *MI*. Transcript abundance is also an important factor because genes suspected of high level expression are likely to exhibit a shift in their codon usage from the species average toward optimal codons selected for translational efficiency.

## Conclusion

Extensive sequence datasets from one complete, one draft, and 30 partial genomes across the phylum Nematoda have been used to analyze the conservation and diversification of encoded protein families [26] and the factors effecting codon usage and bias (the present report). The undertaken comprehensive survey of observed codon usage and bias is based on 26 million codons in 32 species, making it the most extensive study for any phylum. Our data indicate that similarity between species in average codon usage is a short range phenomenon, generally rapidly diminishing beyond the genus level. Mapping codon usage changes to the phyla indicates the genera *Globodera*, *Meloidogyne*, *Pristionchus*, and *Strongyloides *have the most highly derived patterns of codon usage in nematodes, with the remaining species exhibiting less relatively divergence from an ancestral nematode pattern. There was a strong correlation between the exonic GC content and similarity in codon usage. GC content also explains much of the observed variation in the effective number of codons, a measure of codon bias, and even differences in AA frequency. Results from partial genomes assembled from ESTs and complete genomes provide generally good agreement on codon usage, although refinement will be necessary as more sequences become available. EST collections from five species have also been used as a starting point to identify potentially abundant genes and predict optimal codons. These predictions will also be refined using more accurate measures of gene expression, including microarrays and quantitative RT-PCR.

## Materials and methods

### Sequence acquisition and organization

To perform the first meta-analysis of the genomic biology of the phylum Nematoda [26], ESTs from 30 nematode species generated by our laboratories and others were downloaded from the dbEST division of GenBank in May 2003. For consistency, in this accompanying analysis of codon usage we used this dataset for all analyses. Sequences were collated and processed into partial genomes using the PartiGene pipeline [71,72]. Polypeptide translations were predicted using prot4EST [45,72]. Wormpep_dna121 (March 2004; Welcome Trust Sanger Institute, unpublished data) was used for *C. elegans *analysis, and the hybrid gene set [35] was used for *C. briggsae *analysis. Mitochondria can have codon usage differing from that of the nuclear genome, and therefore protein coding genes from mitochondrial genomes were eliminated from consideration. Codon usage tables for human, *Saccharomyces cerevisiae*, and *Escherichia coli *were derived from the Codon Usage Table Database [73] derived from GenBank Release 140.0 (22 March 2004 [74]).

### Phylogenetic correction

Analyses of the relationships among GC content, AA, and codon usage values require statistical correction for the phylogenetic relatedness of the species being studied using phylogenetically independent contrasts [75]. To generate these contrasts, we performed the following procedures. First, to construct a phylogenetic tree independent of the transcriptomic data analyzed in this paper, we aligned 18S ribosomal RNA sequences using Clustalw [76] for all nematodes for which more than 15 kilobases of sequence was available. The 18S sequence GenBank accession numbers are available in Figure 1; the sequences from a priapulid worm and a nematomorph were used as outgroups [16] but excluded from our analysis. Alignments were trimmed to reflect only the overlapping portion of the sequences from all species analyzed. Second, this alignment, containing 1,841 base pairs/species (including gaps) and an alternative alignment excluding any region involving an insertion or deletion (1,423 base pairs/species remained), was used to estimate phylogenies from the nucleotide sequences by parsimony and maximum likelihood (with and without assumption of a molecular clock) using Phylip [77]. Third, the trees with branch lengths derived from molecular clock-based analysis were used to calculate phylogenetically independent contrasts for our parameters of interest [75]. The Phylip program 'contrasts' was used to compute the phylogenetically independent contrasts using a Brownian-motion model [78,79] of genomic evolution.

### Bioinformatics

The Emboss program 'cusp' was used to calculate codon usage in the predicted translations [80]. The ENC [49] was calculated using the Emboss program 'Codon Heterozygosity (Inverse of) in Protein-coding Sequences'. A genetic distance statistic was used to quantify divergence of synonymous codon usage between species [47] follows. Let *t*_*j *_be the number of codons that code for the *j*^th ^amino acid. We omit analysis of the nondegenerate codons Met and Trp, as well as the 'stop' codon, so that *j *= 1, 2 ... *r*, where *r *= 18. Furthermore, let *a*_*ij *_and *b*_*ij *_be the frequencies of the *i*^th ^synonymous codon in the *j*^th ^AA of two organisms A and B, respectively. Then, Nei's difference statistic D is defined as the following:

$\begin{array}{ccc} J_{jaa}=\sum_{i=1}^{t_{j}}a_{ij}^{2} & J_{jbb}=\sum_{i=1}^{t_{j}}b_{ij}^{2} & J_{jab}=\sum_{i=1}^{t_{j}}a_{ij}b_{ij} \end{array}$

$\begin{array}{ccc} J_{aa}=\frac{1}{r}\sum_{j=1}^{r}J_{jaa} & J_{bb}=\frac{1}{r}\sum_{j=1}^{r}J_{jbb} & J_{ab}=\frac{1}{r}\sum_{j=1}^{r}J_{jab} \end{array}$

$D=-\log\left(\frac{J_{ab}}{\sqrt{J_{aa}J_{bb}}}\right).$

Investigators have used D as an empirical measure of difference, averaged over all *r *residues, of the codon usage between organisms [48]. There are a total of C_32,2 _= 496 meaningful comparisons for the entire collection of 32 species. These results are presented as an N × N square matrix and the values are presented as D × 100. For simplicity in the remainder of the text we will refer to D × 100 as D_100_.

Phylogenetic changes in codon usage were analyzed using the species tree derived from 18S rRNA sequences estimated by maximum likelihood with a molecular clock imposed. Partitioning a matrix of distance values on a phylogenetic tree can estimate amounts of change occurring on each branch, provided that the distance metrics obey the triangle inequality (see the discussion on page 25 of the report by Page and Holmes [81]). Because of its logarithmic operation, Nei's difference statistic D violates the triangle inequality at high values. For the phylogenetic analysis of codon usage, we substituted for D a distance measure equal to 1 - antilog(-D), which obeys the triangle inequality. Distances were partitioned on the tree topology using the cyclic neighbor-joining algorithm illustrated by Avise [82], except that the topology was specified by the prior analysis of 18S rRNA sequences.

The ENC was used to measure the degree of codon bias for a gene [49]. Because the ENC statistic is not reliable when analyzing very short sequences (20 AAs or less), 54 short translations out of a total of 70,358 were discarded from these analyses. The relative abundance of nematode codons (per species) having a statistically significant N1 context-dependent codon bias was calculated by computing the R values and the standard deviations, as described by Fedorov and coworkers [57].

Predicted expression level of a transcript (abundant, moderate, rare, and so on) was determined by counting the number of ESTs comprising the cluster. Five species from different clades that have been sampled with at least 10,000 ESTs from several life-stage libraries were selected for these analyses. Most of the cDNA libraries were constructed using the same protocols [61,83], and although the libraries generally correlate with abundance in the original biologic sample, artifacts can occur. The increase in use of a given codon for an AA in highly expressed genes (optimal codons) was considered significant when the difference of the codon distributions within that AA was statistically significant between datasets (*P *≤ 0.01).

To assess the differences in calculated codon usage distributions when using partial (EST-based) as compared with whole genome data, we generated two datasets using *C. elegans *ESTs and compared them with the curated gene set of *C. elegans *(Wormpep version 121). Each EST dataset was composed of 10,000 ESTs (approximately the average number of ESTs used for the other 30 species); clustering and peptide predictions were performed using the same algorithms as for the other species.

## Additional data files

The following additional data are included with the online version of this article: An Excel file containing a table that shows the nucleotide usage (%) by codon position and nematode species (Additional data file 1); an Excel file containing an N × N square matrix that shows codon usage across degenerate AAs for 25 nematode species reported as D values (Additional data file 2); a PowerPoint file containing a figure that shows the distribution of genes with various degrees of codon usage bias as measured by ENC for three species with approximately the same number of clusters but with different coding GC content (*S. ratti *[GC = 32%], *P. trichosuri *[40%], and *P. pacificus *[51%]; Additional data file 3); an Excel file containing a table that shows the N1 context dependent bias per species (Additional data file 4); and an Excel file containing a table that shows codon usage of abundant and less abundant translations for five nematode species (Additional data file 5).

## Supplementary Material

Additional file 1 Click here for file

Additional file 2 Click here for file

Additional file 3 Click here for file

Additional file 4 Click here for file

Additional file 5 Click here for file

## References

[B1] Milhon JL Tracy JW Updated codon usage in *Schistosoma*. Exp Parasitol 1995 80 353 356 10.1006/expr.1995.1046 7895849

[B2] Duret L Evolution of synonymous codon usage in metazoans. Curr Opin Genet Dev 2002 12 640 649 10.1016/S0959-437X(02)00353-2 12433576

[B3] Sueoka N On the genetic basis of variation and heterogeneity of DNA base composition. Proc Natl Acad Sci USA 1962 48 582 592 10.1073/pnas.48.4.582 13918161 PMC220819

[B4] Sueoka N Directional mutation pressure and neutral molecular evolution. Proc Natl Acad Sci USA 1988 85 2653 2657 10.1073/pnas.85.8.2653 3357886 PMC280056

[B5] Knight RD Freeland SJ Landweber LF A simple model based on mutation and selection explains trends in codon and amino-acid usage and GC composition within and across genomes. Genome Biol 2001 2 research0010 11305938 10.1186/gb-2001-2-4-research0010 PMC31479

[B6] Chen SL Lee W Hottes AK Shapiro L McAdams HH Codon usage between genomes is constrained by genome-wide mutational processes. Proc Natl Acad Sci USA 2004 101 3480 3485 10.1073/pnas.0307827100 14990797 PMC373487

[B7] Ikemura T Codon usage and tRNA content in unicellular and multicellular organisms. Mol Biol Evol 1985 2 13 34 3916708 10.1093/oxfordjournals.molbev.a040335

[B8] Moriyama EN Powell JR Codon usage bias and tRNA abundance in *Drosophila*. J Mol Evol 1997 45 514 523 10.1007/PL00006256 9342399

[B9] Bulmer M Coevolution of codon usage and transfer RNA abundance. Nature 1987 325 728 730 10.1038/325728a0 2434856

[B10] Sharp PM Li WH The codon Adaptation Index: a measure of directional synonymous codon usage bias, and its potential applications. Nucleic Acids Res 1987 15 1281 1295 3547335 10.1093/nar/15.3.1281 PMC340524

[B11] Gouy M Gautier C Codon usage in bacteria: correlation with gene expressivity. Nucleic Acids Res 1982 10 7055 7074 6760125 10.1093/nar/10.22.7055 PMC326988

[B12] Carlini DB Chen Y Stephan W The relationship between third-codon position nucleotide content, codon bias, mRNA secondary structure and gene expression in the drosophilid alcohol dehydrogenase genes Adh and Adhr. Genetics 2001 159 623 633 11606539 10.1093/genetics/159.2.623 PMC1461829

[B13] Chamary JV Hurst LD Evidence for selection on synonymous mutations affecting stability of mRNA secondary structure in mammals. Genome Biol 2005 6 R75 10.1186/gb-2005-6-9-r75 16168082 PMC1242210

[B14] Oresic M Dehn M Korenblum D Shalloway D Tracing specific synonymous codon-secondary structure correlations through evolution. J Mol Evol 2003 56 473 484 10.1007/s00239-002-2418-x 12664167

[B15] Marquez R Smit S Knight R Do universal codon-usage patterns minimize the effects of mutation and translation error? Genome Biol 2005 6 R91 10.1186/gb-2005-6-11-r91 16277746 PMC1297647

[B16] Blaxter ML De Ley P Garey JR Liu LX Scheldeman P Vierstraete A Vanfleteren JR Mackey LY Dorris M Frisse LM A molecular evolutionary framework for the phylum Nematoda. Nature 1998 392 71 75 10.1038/32160 9510248

[B17] The C. elegans Sequencing Consortium Genome sequence of the nematode *C. elegans *: a platform for investigating biology. Science 1998 282 2012 2018 10.1126/science.282.5396.2012 9851916

[B18] Blaxter ML Raghavan N Ghosh I Guiliano D Lu W Williams SA Slatko B Scott AL Genes expressed in *Brugia malayi *infective third stage larvae. Mol Biochem Parasitol 1996 77 77 93 10.1016/0166-6851(96)02571-6 8784774

[B19] Dautova M Rosso MN Abad P Gommers FJ Bakker J Smant G Single pass cDNA sequencing - a powerful tool to analyse gene expression in preparasitic juveniles of the southern root-knot nematode *Meloidogyne incognita*. Nematology 2001 3 129 139 10.1163/156854101750236259

[B20] McCarter J Dautova Mitreva M Martin J Dante M Wylie T Rao U Pape D Bowers Y Theising B Murphy CV Analysis and functional classification of transcripts from the Nematode *Meloidogyne incognita*. Genome Biol 2003 4 R26 10.1186/gb-2003-4-4-r26 12702207 PMC154577

[B21] Daub J Loukas A Pritchard DI Blaxter M A survey of genes expressed in adults of the human hookworm, *Necator americanus*. Parasitology 2000 120 171 184 10.1017/S0031182099005375 10726278

[B22] Mitreva M McCarter JP Martin J Dante M Wylie T Chiapelli B Pape D Clifton SW Nutman TB Waterston RH Comparative genomics of gene expression in the parasitic and free-living nematodes *Strongyloides stercoralis *and *Caenorhabditis elegans*. Genome Res 2004 14 209 220 10.1101/gr.1524804 14762059 PMC327096

[B23] Thompson FJ Mitreva M Barker GL Martin J Waterston RH McCarter JP Viney ME An expressed sequence tag analysis of the life-cycle of the parasitic nematode *Strongyloides ratti*. Mol Biochem Parasitol 2005 142 32 46 10.1016/j.molbiopara.2005.03.006 15907559

[B24] Parkinson J Mitreva M Hall N Blaxter M McCarter JP 400000 nematode ESTs on the Net. Trends Parasitol 2003 19 283 286 10.1016/S1471-4922(03)00132-6 12855373

[B25] Mitreva M Blaxter ML Bird DM McCarter JP Comparative genomics of nematodes. Trends Genet 2005 21 573 581 10.1016/j.tig.2005.08.003 16099532

[B26] Parkinson J Mitreva M Whitton C Thomson M Daub J Martin J Hall N Barrell B Waterston RH McCarter JP A transcriptomic analysis of the phylum Nematoda. Nat Genet 2004 36 1259 1267 10.1038/ng1472 15543149

[B27] Nematode Net http://www.nematode.net/

[B28] Wylie T Martin J Dante M Mitreva M Clifton SW Chinwalla A Waterston RH Wilson RK McCarter JP Nematode.net: a tool for navigating sequences from parasitic and free-living nematodes. Nucleic Acids Res 2004 32 D423 D426 10.1093/nar/gkh010 14681448 PMC308745

[B29] Nembase http://www.nematodes.org/

[B30] Parkinson J Whitton C Schmid R Thomson M Blaxter M NEMBASE: a resource for parasitic nematode ESTs. Nucleic Acids Res 2004 32 D427 D430 10.1093/nar/gkh018 14681449 PMC308753

[B31] Stenico M Lloyd AT Sharp PM Codon usage in *Caenorhabditis elegans *: delineation of translational selection and mutational biases. Nucleic Acids Res 1994 22 2437 2446 8041603 10.1093/nar/22.13.2437 PMC308193

[B32] Duret L Mouchiroud D Expression pattern and, surprisingly, gene length shape codon usage in *Caenorhabditis*, *Drosophila*, and *Arabidopsis*. Proc Natl Acad Sci USA 1999 96 4482 4487 10.1073/pnas.96.8.4482 10200288 PMC16358

[B33] Duret L tRNA gene number and codon usage in the *C. elegans *genome are co-adapted for optimal translation of highly expressed genes. Trends Genet 2000 16 287 289 10.1016/S0168-9525(00)02041-2 10858656

[B34] Marais G Duret L Synonymous codon usage, accuracy of translation, and gene length in *Caenorhabditis elegans*. J Mol Evol 2001 52 275 280 11428464 10.1007/s002390010155

[B35] Stein LD Bao Z Blasiar D Blumenthal T Brent MR Chen N Chinwalla A Clarke L Clee C Coghlan A The genome sequence of *Caenorhabditis briggsae *: a platform for comparative genomics. PLoS Biol 2003 1 E45 10.1371/journal.pbio.0000045 14624247 PMC261899

[B36] Unnasch TR Katholi CR Coate LM *Onchocerca volvulus *: frequency of codon usage. Exp Parasitol 1992 75 457 459 10.1016/0014-4894(92)90259-D 1493878

[B37] Hammond MP Codon usage and gene organization in *Brugia*. Parasitol Res 1994 80 173 175 10.1007/BF00933788 8202460

[B38] Ellis J Morrison DA Kalinna B Comparison of the patterns of codon usage and bias between *Brugia*, *Echinococcus*, *Onchocerca *and *Schistosoma *species. Parasitol Res 1995 81 388 393 10.1007/BF00931499 7501637

[B39] Fadiel A Lithwick S Wanas MQ Cuticchia AJ Influence of intercodon and base frequencies on codon usage in filarial parasites. Genomics 2001 74 197 210 10.1006/geno.2001.6531 11386756

[B40] Moore TA Ramachandran S Gam AA Neva FA Lu W Saunders L Williams SA Nutman TB Identification of novel sequences and codon usage in *Strongyloides stercoralis*. Mol Biochem Parasitol 1996 79 243 248 10.1016/0166-6851(96)02659-X 8855562

[B41] Fadiel AA Lithwick S Gamra MM Codon usage analysis of *Ascaris *species influence of base and intercodon frequencies on the synonymous codon usage. J Egypt Soc Parasitol 2002 32 625 638 12214939

[B42] Fadiel AA Lithwick S el-Garhy MF Influence of parasitic life style on the patterns of codon usage and base frequencies of *Ancylostoma *and *Necator *species. J Egypt Soc Parasitol 2002 32 657 673 12214942

[B43] Nakamura Y Gojobori T Ikemura T Codon usage tabulated from the international DNA sequence databases; its status 1999. Nucleic Acids Res 1999 27 292 10.1093/nar/27.1.292 9847205 PMC148160

[B44] McCarter JP Bird DM Mitreva M Nematode gene sequences: update for December 2005. J Nematol 2006 37 417 421 19262885 PMC2620984

[B45] Wasmuth JD Blaxter ML prot4EST: translating expressed sequence tags from neglected genomes. BMC Bioinformatics 2004 5 187 10.1186/1471-2105-5-187 15571632 PMC543579

[B46] King JL Jukes TH Non-Darwinian evolution. Science 1969 164 788 798 5767777 10.1126/science.164.3881.788

[B47] Nei M Genetic distance between populations. Am Naturalist 1972 106 283 292 10.1086/282771

[B48] Long M Gillespie JH Codon usage divergence of homologous vertebrate genes and codon usage clock. J Mol Evol 1991 32 6 15 10.1007/BF02099923 1901369

[B49] Wright F The 'effective number of codons' used in a gene. Gene 1990 87 23 29 10.1016/0378-1119(90)90491-9 2110097

[B50] Powell JR Moriyama EN Evolution of codon usage bias in *Drosophila*. Proc Natl Acad Sci USA 1997 94 7784 7790 10.1073/pnas.94.15.7784 9223264 PMC33704

[B51] Comeron JM Aguade M An evaluation of measures of synonymous codon usage bias. J Mol Evol 1998 47 268 274 10.1007/PL00006384 9732453

[B52] Yarus M Folley LS Sense codons are found in specific contexts. J Mol Biol 1984 182 529 540 10.1016/0022-2836(85)90239-6 3892014

[B53] Shpaer EG Constraints on codon context in *Escherichia coli *genes. Their possible role in modulating the efficiency of translation. J Mol Biol 1986 188 555 564 10.1016/S0022-2836(86)80005-5 3525848

[B54] Gouy M Codon contexts in *Enterobacterialand coliphage *genes. Mol Biol Evol 1987 4 426 444 3128715 10.1093/oxfordjournals.molbev.a040450

[B55] Berg OG Silva PJN Codon bias in *Escherichia coli*: the influence of codon context on mutationand selection. Nucleic Acids Res 1997 25 1397 1404 10.1093/nar/25.7.1397 9060435 PMC146607

[B56] Karlin S Mrazek J What drives codon choices in human genes? J Mol Biol 1996 262 459 472 10.1006/jmbi.1996.0528 8893856

[B57] Fedorov A Saxonov S Gilbert W Regularities of context-dependent codon bias in eukaryotic genes. Nucleic Acids Res 2002 30 1192 1197 10.1093/nar/30.5.1192 11861911 PMC101244

[B58] Eyre-Walker A Hurst LD The evolution of isochores. Nat Rev Genet 2001 2 549 555 10.1038/35080577 11433361

[B59] Audic S Claverie JM The significance of digital gene expression profiles. Genome Res 1997 7 986 995 9331369 10.1101/gr.7.10.986

[B60] Munoz ET Bogarad LD Deem MW Microarray and EST database estimates of mRNA expression levels differ: the protein length versus expression curve for *C. elegans*. BMC Genomics 2004 5 30 10.1186/1471-2164-5-30 15134588 PMC434498

[B61] Mitreva M Jasmer DP Appleton J Martin J Dante M Wylie T Clifton SW Waterston RH McCarter JP Gene discovery in the adenophorean nematode *Trichinella spiralis*: an analysis of transcription from three life cycle stages. Mol Biochem Parasitol 2004 137 277 291 10.1016/j.molbiopara.2004.05.015 15383298

[B62] Ikemura T Correlation between the abundance of *Escherichia coli *transfer RNAs and the occurrence of the respective codons in its protein genes: a proposal for a synonymous codon choice that is optimal for the *E. coli *translational system. J Mol Biol 1981 151 389 409 10.1016/0022-2836(81)90003-6 6175758

[B63] Musto H Romero H Zavala A Jabbari K Bernardi G Synonymous codon choices in the extremely GC-poor genome of *Plasmodium falciparum*: compositional constraints and translational selection. J Mol Evol 1999 49 27 35 10.1007/PL00006531 10368431

[B64] Kanaya S Yamada Y Kinouchi M Kudo Y Ikemura T Codon usage and tRNA genes in eukaryotes: correlation of codon usage diversity with translation efficiency and with CG-dinucleotide usage as assessed by multivariate analysis. J Mol Evol 2001 53 290 298 10.1007/s002390010219 11675589

[B65] Ohama T Yamao F Muto A Osawa S Organization and codon usage of the streptomycin operon in *Micrococcus luteus*, a bacterium with a high genomic G + C content. J Bacteriol 1987 169 4770 4777 3654584 10.1128/jb.169.10.4770-4777.1987 PMC213853

[B66] Ohama T Muto A Osawa S Role of GC-biased mutation pressure on synonymous codon choice in *Micrococcus luteus*, a bacterium with a high genomic GC-content. Nucleic Acids Res 1990 18 1565 1569 2326195 10.1093/nar/18.6.1565 PMC330526

[B67] Sueoka N Directional mutation pressure, selective constraints, and genetic equilibria. J Mol Evol 1992 35 95 114 1556753 10.1007/BF00182387

[B68] Wilquet V Van de Casteele M The role of the codon first letter in the relationship between genomic GC content and protein amino acid composition. Res Microbiol 1999 150 21 32 10.1016/S0923-2508(99)80043-6 10096131

[B69] D'Onofrio G Mouchiroud D Aïssani B Gautier C Bernardi G Correlations between the compositional properties of human genes, codon usage, and amino acid composition of proteins. J Mol Evol 1991 32 504 510 10.1007/BF02102652 1908021

[B70] Lobry JR Influence of genomic G+C content on average amino-acid composition of proteins from 59 bacterial species. Gene 1997 205 309 316 10.1016/S0378-1119(97)00403-4 9461405

[B71] Parkinson J Guiliano DB Blaxter M Making sense of EST sequences by CLOBBing them. BMC Bioinformatics 2002 3 31 10.1186/1471-2105-3-31 12398795 PMC137596

[B72] Parkinson J Anthony A Wasmuth J Schmid R Hedley A Blaxter M PartiGene: constructing partial genomes. Bioinformatics 2004 20 1398 1404 10.1093/bioinformatics/bth101 14988115

[B73] Codon Usage Table Database http://www.kazusa.or.jp/codon/

[B74] Nakamura Y Gojobori T Ikemura T Codon usage tabulated from international DNA sequence databases: status for the year 2000. Nucleic Acids Res 2000 28 292 10.1093/nar/28.1.292 10592250 PMC102460

[B75] Felsenstein J Phylogenies and the comparative method. Am Naturalist 1985 125 1 12 10.1086/284325 31094602

[B76] Chenna R Sugawara H Koike T Lopez R Gibson TJ Higgins DG Thompson JD Multiple sequence alignment with the Clustal series of programs. Nucleic Acids Res 2003 31 3497 3500 10.1093/nar/gkg500 12824352 PMC168907

[B77] Felsenstein J PHYLIP - Phylogeny Inference Package (Version 3.2). Cladistics 1989 5 164 166

[B78] Cavalli-Sforza LL Edwards AWF Phylogenetic analysis: models and estimation procedures. Evolution 1967 32 550 570 10.2307/2406616 28563688

[B79] Edwards AWF Cavalli-Sforza LL Heywood VH, McNeill Reconstruction of evolutionary trees. Phenetic and Phylogenetic Classification 1964 6 London: Systematics Association 67 76

[B80] Rice P Longden I Bleasby A EMBOSS: the European Molecular Biology Open Software Suite. Trends Genet 2000 16 276 277 10.1016/S0168-9525(00)02024-2 10827456

[B81] Page RDM Holmes EC Molecular Evolution: a Phylogenetic Approach 1998 Oxford, UK: Blackwell Science

[B82] Avise JC Molecular Markers Natural History and Evolution 1994 New York: Chapman and Hall

[B83] Mitreva M Elling AA Dante M Kloek AP Kalyanaraman A Aluru S Clifton SW Bird DM Baum TJ McCarter JP A survey of SL1-spliced transcripts from the root-lesion nematode *Pratylenchus penetrans*. Mol Gen Genomics 2004 272 138 148 10.1007/s00438-004-1054-0 15338281

