# Supplementary figures and images for: Codon usage patterns in Nematoda: analysis based on over 25 million codons in thirty-two species
Source: Genome Biol. 2006 Aug 14;7(8):R75. doi: 10.1186/gb-2006-7-8-r75 (PMC1779591; doi:10.1186/gb-2006-7-8-r75)

## Slide 1
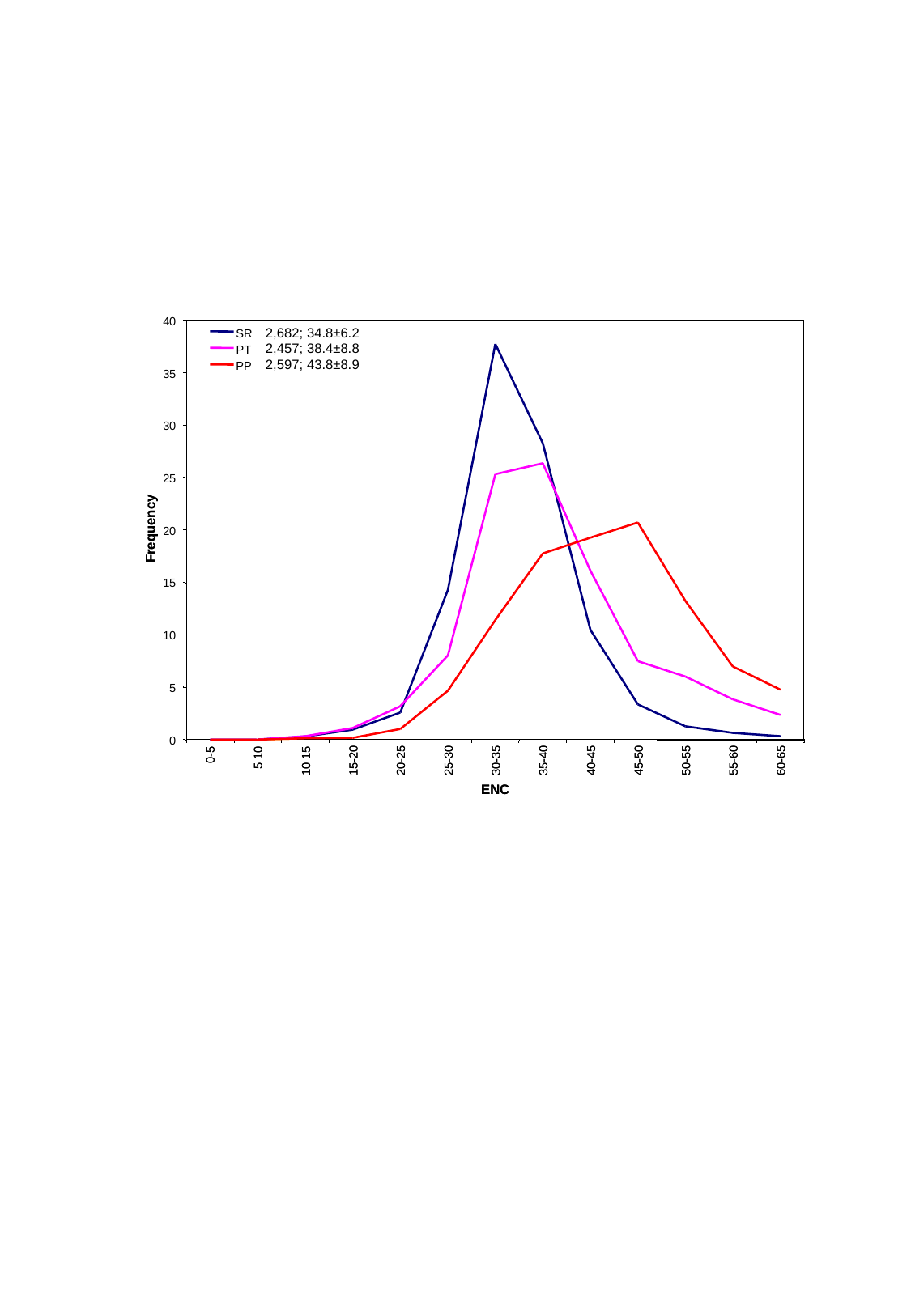

40
(2,682; 34.8
2,682; 34.8±6.2
±
6.2)
SR
SR
(2,457; 38.4
2,457; 38.4±8.8
±
8.8)
PT
PT
(2,597; 43.8
2,597; 43.8±8.9
±
8.9)
PP
PP
35
30
25
Frequency
Frequency
20
15
10
5
0
0-5
0-5
5 10
5 10
15-20
15-20
20-25
20-25
25-30
25-30
30-35
30-35
35-40
35-40
40-45
40-45
45-50
45-50
50-55
50-55
55-60
55-60
60-65
60-65
10 15
10 15
ENC
ENC

Supplement: Additional file 3 [file gb-2006-7-8-r75-S3.ppt]
